# Supplementary figures and images for: Colonization with extended spectrum beta-lactamase and carbapenemases producing Enterobacteriaceae among hospitalized patients at the global level: A systematic review and meta-analysis
Source: PLoS One. 2023 Nov 27;18(11):e0293528. doi: 10.1371/journal.pone.0293528 (PMC10681255; doi:10.1371/journal.pone.0293528)

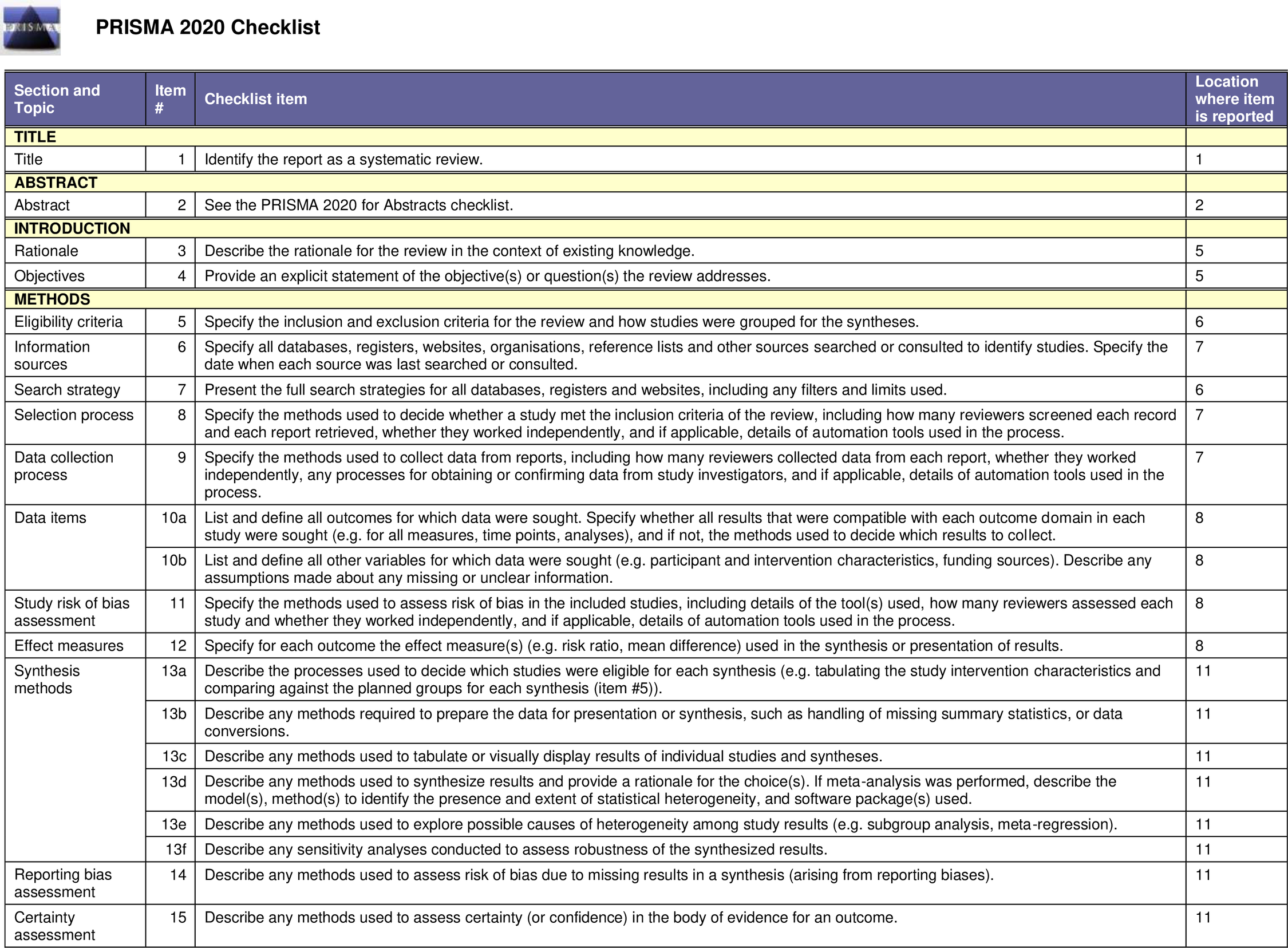

Supplement: S1 Checklist — (TIF) [file pone.0293528.s001.tif]
