# Supplementary material for: Colonization with extended spectrum beta-lactamase and carbapenemases producing Enterobacteriaceae among hospitalized patients at the global level: A systematic review and meta-analysis
Source: PLoS One. 2023 Nov 27;18(11):e0293528. doi: 10.1371/journal.pone.0293528 (PMC10681255; doi:10.1371/journal.pone.0293528)
Supplement: Attachment — Submitted filename: 1st assignment-PONE-D-23-02816 after revision, 2023.pdf [file pone.0293528.s002.pdf]

PLOS ONE

Colonization with exteneded spectrum beta-lactamase and Carbapenem producing Enterobacteriacae among hospitalized patients at the global level: a systematic review and meta-analysis.

--Manuscript Draft--

**Manuscript Number:**	PONE-D-23-02816
**Article Type:**	Research Article
**Full Title:**	Colonization with exteneded spectrum beta-lactamase and Carbapenem producing Enterobacteriacae among hospitalized patients at the global level: a systematic review and meta-analysis.
**Short Title:**	Colonization with extended spectrum beta-lactamase and Carbapenem producing Enterobacteriaceae.
**Corresponding Author:**	Dessie Abera Aefera, Msc<br>Addis Ababa University College of Health Sciences<br>Addis Ababa, Addis Ababa ETHIOPIA
**Keywords:**	Colonization rate; ESBL; CRE
**Abstract:**	Background<br><br>Gut commensal bacteria can mediate resistance against pathogenic bacteria. However, exposure to antibiotics and hospitalization may facilitate the emergence of multidrug resistant bacteria. We aimed to provide comprehensive evidence about colonization rate of extended spectrum beta-lactamase and carbapenem producing Enterobacteriaceae.<br><br>Method<br><br>We used PubMed, Google Scholar and Web Sciences data bases to search studies from January 1, 2016 to August10, 2022 about colonization rate of extended spectrum beta-lactamase and carbapenem producing Enterobacteriaceae. Data were extracted from eligible studies and analyzed using Stata version 16 software. The quality of included studies was assessed using the Joanna Briggs Institute Critical Appraisal tools.<br><br>Results<br><br>We identified 342 studies from the comprehensive data search and data were extracted from 20 studies. The pooled estimate of extended spectrum beta-lactamase and carbapenem producing Enterobacteriaceae were 45.6%(95%CI: 34.11-57-10) and 16.19% (95% CI: 5.46-26.91) respectively. The predominant extended spectrum beta-lactamase producers were E.coli,32.99% (95% CI: 23.28-42.69) and K.pneumoniae, 11.43% (95% CI:7.98-14.89). Prolonged hospitalization was linked to carbapenem producing Enterobacteriaceae colonization with the odds of 14.77 (95% CI: -1.35-30.90) at admission and 45.63 (95% CI: 0.86-92.12) after ≥7 days of admission.<br><br>Conclusion<br><br>In this study, significant colonization rate of extended spectrum beta-lactamase and carbapenem producing Enterobacteriaceae were determined. Previous antibiotic consumption and prolonged hospitalization were associated with the colonization of extended spectrum beta-lactamase and carbapenem producing Enterobacteriaceae. This indicates the need for strong mitigation strategies to minimize the spread of multidrug-resistant bacteria at the healthcare facilities.
**Order of Authors:**	Dessie Abera Tefera, Msc
	Ayinalem Alemu, MSc
	Adane Mihret, PhD

Powered by Editorial Manager® and ProduXion Manager® from Aries Systems Corporation

	Abel Abera Negash, PhD
	Woldeaeregay Erku Abegaze, PhD
	Ken Cadwell, PhD
**Additional Information:**	
**Question**	**Response**
**Financial Disclosure**<br><br>Enter a financial disclosure statement that describes the sources of funding for the work included in this submission. Review the submission guidelines for detailed requirements. View published research articles from *PLOS ONE* for specific examples.<br><br>This statement is required for submission and **will appear in the published article** if the submission is accepted. Please make sure it is accurate.<br><br>**Unfunded studies**<br>Enter: *The author(s) received no specific funding for this work.*<br><br>**Funded studies**<br>Enter a statement with the following details:<br>• Initials of the authors who received each award<br>• Grant numbers awarded to each author<br>• The full name of each funder<br>• URL of each funder website<br>• Did the sponsors or funders play any role in the study design, data collection and analysis, decision to publish, or preparation of the manuscript?<br>• **NO** - Include this sentence at the end of your statement: *The funders had no role in study design, data collection and analysis, decision to publish, or preparation of the manuscript.*<br>• **YES** - Specify the role(s) played.<br><br>* typeset	The authors received no specific funding for this work.
**Competing Interests**<br><br>Use the instructions below to enter a competing interest statement for this submission. On behalf of all authors, disclose any competing interests that could be perceived to bias this	The authors have declared that no competing interests exist.

work—acknowledging all financial support and any other relevant financial or non-financial competing interests.<br><br>This statement is required for submission and **will appear in the published article** if the submission is accepted. Please make sure it is accurate and that any funding sources listed in your Funding Information later in the submission form are also declared in your Financial Disclosure statement.<br><br>View published research articles from *PLOS ONE* for specific examples.<br><br>**NO authors have competing interests**<br><br>Enter: *The authors have declared that no competing interests exist.*<br><br>**Authors with competing interests**<br><br>Enter competing interest details beginning with this statement:<br><br>*I have read the journal's policy and the authors of this manuscript have the following competing interests: [insert competing interests here]*<br><br>* typeset	
**Ethics Statement**<br><br>Enter an ethics statement for this submission. This statement is required if the study involved:<br><br>• Human participants<br>• Human specimens or tissue<br>• Vertebrate animals or cephalopods<br>• Vertebrate embryos or tissues<br>• Field research<br><br>Write "N/A" if the submission does not require an ethics statement.	N/A

General guidance is provided below. Consult the submission guidelines for detailed instructions. **Make sure that all information entered here is included in the Methods section of the manuscript.**<br><br>**Format for specific study types**<br><br>**Human Subject Research (involving human participants and/or tissue)**<br>• Give the name of the institutional review board or ethics committee that approved the study<br>• Include the approval number and/or a statement indicating approval of this research<br>• Indicate the form of consent obtained (written/oral) or the reason that consent was not obtained (e.g. the data were analyzed anonymously)<br><br>**Animal Research (involving vertebrate animals, embryos or tissues)**<br>• Provide the name of the Institutional Animal Care and Use Committee (IACUC) or other relevant ethics board that reviewed the study protocol, and indicate whether they approved this research or granted a formal waiver of ethical approval<br>• Include an approval number if one was obtained<br>• If the study involved *non-human primates*, add *additional details* about animal welfare and steps taken to ameliorate suffering<br>• If anesthesia, euthanasia, or any kind of animal sacrifice is part of the study, include briefly which substances and/or methods were applied<br><br>**Field Research**<br><br>Include the following details if this study involves the collection of plant, animal, or other materials from a natural setting:<br>• Field permit number<br>• Name of the institution or relevant body that granted permission	
**Data Availability**<br><br>Authors are required to make all data underlying the findings described fully	Yes - all data are fully available without restriction

Powered by Editorial Manager® and ProduXion Manager® from Aries Systems Corporation

available, without restriction, and from the time of publication. PLOS allows rare exceptions to address legal and ethical concerns. See the PLOS Data Policy and FAQ for detailed information.<br><br>A Data Availability Statement describing where the data can be found is required at submission. Your answers to this question constitute the Data Availability Statement and **will be published in the article**, if accepted.<br><br>**Important:** Stating 'data available on request from the author' is not sufficient. If your data are only available upon request, select 'No' for the first question and explain your exceptional situation in the text box.<br><br>Do the authors confirm that all data underlying the findings described in their manuscript are fully available without restriction?	
**Describe where the data may be found in full sentences. If you are copying our sample text, replace any instances of XXX with the appropriate details.**<br><br>• If the data are **held or will be held in a public repository**, include URLs, accession numbers or DOIs. If this information will only be available after acceptance, indicate this by ticking the box below. For example: *All XXX files are available from the XXX database (accession number(s) XXX, XXX.*).<br>• If the data are all contained **within the manuscript and/or Supporting Information files**, enter the following: *All relevant data are within the manuscript and its Supporting Information files.*<br>• If neither of these applies but you are able to provide **details of access elsewhere**, with or without limitations, please do so. For example:<br><br>*Data cannot be shared publicly because of [XXX]. Data are available from the XXX Institutional Data Access / Ethics*	All relevant data are within the manuscript and its supporting information files.

Powered by Editorial Manager® and ProduXion Manager® from Aries Systems Corporation

Committee (contact via XXX) for researchers who meet the criteria for access to confidential data.<br><br>*The data underlying the results presented in the study are available from (include the name of the third party and contact information or URL).*<br>• This text is appropriate if the data are owned by a third party and authors do not have permission to share the data.<br><br>* typeset	
Additional data availability information:	Tick here if the URLs/accession numbers/DOIs will be available only after acceptance of the manuscript for publication so that we can ensure their inclusion before publication.

Powered by Editorial Manager® and ProduXion Manager® from Aries Systems Corporation

Manuscript

Click here to access/download;Manuscript;Systematic review-plos one.docx

The word Carbapenem must be replace by Carbapenemases producing.....

Colonization with exteneded spectrum beta-lactamase and Carbapenem producing *Enterobacteriacae* among hospitalized patients at the global level: a systematic review and meta-analysis.

Dessie Abera*[1,2], Ayinalem Alemu[3,4], Adane Mihret[2], Abel Abera Negash[2], Woldeaeregay Erku Abegaze[2], Ken Cadwell[5,6]

[1] Department of Medical Laboratory Sciences, College of Health Sciences, Addis Ababa University, Addis Ababa, Ethiopia.

[2]Department of Microbiology, Immunology and Parasitology, College of Health Sciences, Addis Ababa University, Addis Ababa, Ethiopia.

[3]Ethiopian Public Health Institute, Addis Ababa, Ethiopia.

[4]Aklilu Lemma Institute of Pathobiology, Addis Ababa University, Addis Ababa, Ethiopoia.

[5] Department of Microbiology, New York University Grossman School of Medicine, New York, NY 10016, USA

[6] Division of Gastroenterology and Hepatology, Department of Medicine, New York University Langone Health, New York, NY 10016, USA

*Corresponding Author: Dessie Abera

E-mail: dessabera@gmail.com

Addis Ababa University

Phone:+251911993530

Abstract

Background: Gut commensal bacteria can mediate resistance against pathogenic bacteria. However, exposure to antibiotics and hospitalization may facilitate the emergence of multidrug resistant bacteria. We aimed to conduct a systematic review and meta-analysis to provide comprehensive evidence about colonization rate of extended spectrum beta-lactamase and carbapenem producing *Enterobacteriaceae*.

mention carbapenemases instead of carbapenem to describe the study enzymes uniformly.

Method: We used PubMed, Google Scholar and Web Sciences data bases to search studies from January 1, 2016 to August10, 2022 about colonization rate of extended spectrum beta-lactamase and carbapenem producing *Enterobacteriaceae*. Data were extracted from eligible studies and analyzed using Stata version 16 software. The quality of included studies was assessed using the Joanna Briggs Institute Critical Appraisal tools. Random-effects model was used for the analysis, and heterogeneity analysis and publication bias test were conducted.

Results: We identified 342 studies from the comprehensive data search and data were extracted from 20 studies. The pooled estimate of extended spectrum beta-lactamase and carbapenem producing *Enterobacteriaceae* were 45.6%(95%CI: 34.11-57-10) and 16.19% (95% CI: 5.46-26.91) respectively. The predominant extended spectrum beta-lactamase producers were *E.coli*,32.99% (95% CI: 23.28-42.69) and *K.pneumoniae*, 11.43% (95% CI:7.98-14.89). Prolonged hospitalization was linked to carbapenem producing *Enterobacteriaceae* colonization with the odds of 14.77 (95% CI: -1.35-30.90) at admission and 45.63 (95% CI: 0.86-92.12) after ≥7 days of admission.

Conclusion: In this study, significant colonization rate of extended spectrum beta-lactamase and carbapenem producing *Enterobacteriaceae* were determined. Previous antibiotic consumption and

prolonged hospitalization were associated with the colonization of extended spectrum beta-lactamase and carbapenem producing *Enterobacteriaceae*. However, some limitations were also identified. The finding has indicated the need for strong mitigation strategies to minimize the spread of multidrug-resistant bacteria at the healthcare facilities.

Keywords: Colonization rate, ESBL,CRE CRE is deleted and replace by MDRB

1. Introduction

The human gut contains a myriad of commensal bacteria that can mediate resistance (1). However, exposure to antibiotics may reduce the number and function as well as exert selection pressure on the gut microbiome which may promote the emergence of multidrug-resistant bacteria (2). In particular, hospitalized patients are at increased risk of developing infections because of their lower immune status and occurrence of multidrug-resistant bacteria (MDRB) (3). The emergence of MDRB in combination with altered gut microbiome may complicate patients' outcome (4). Gram-negative bacteria, especially *Enterobacteriaceae* are frequently reported in the hospital settings. These bacteria are well known in producing extended-spectrum beta-lactamases (ESBLs) and carbapenemase. *E. coli* and *K. pneumoniae,* in particular, have increased rapidly at the global level in both hospital and community settings (5, 6).

The mechanisms how MDRB emerge in the hospital settings are complicated, but few mechanisms have been proposed. The first way is, resistant bacteria may get into the hospital at the time of admission of patients who already have colonized with resistant strain. Secondly, during prolonged hospitalization, susceptible bacteria may acquire resistant gene through conjugation mechanism or through genetic mutation (7). There are many factors that can facilitate the colonization rate of MDRB in hospitalized patients: such as low hygiene practice, improper antibiotic usage, prolonged hospitalization, implanted materials and sharing a single room with high carrier patients. In addition, patients may remain colonized by MDRB after they leave the hospital and become a potential source of resistant gene transmission for the community (8).

Despite the improvement of using antimicrobial stewardship including personal and environment hygiene, colonization with multi drug-resistant bacteria in the hospitalized patients is still a global challenge (9). Therefore, screening MDRB carriage of patients prior to admission, during

admission and before discharge is critical for mitigating the transmission of resistant bacteria from hospital to community and community to hospital settings (10). Isolation room for MDRB carrier patients is also another recommended mechanism to prevent transmission of resistant bacteria, but lack of resource may limit the widespread use of this approach (11). Many studies have been conducted on colonization of MDRB from multiple sample sources by focusing on a specific geographical location (12-15). However, there is limited information whether there are factors that determine colonization with extended spectrum beta-lactamase producing Enterobacteriaceae (ESBL-PE) and carabapenem resistant Enterobacteriaceae (CRE) that are true across distinct geographic regions. Therefore, the current systematic review and meta-analysis aimed to assess colonization rate and risk factors of ESBL-PE and CRE colonization among hospitalized patients at the global level.

The following references must be used to support the idea:-

Al-Qaysi, A. K., Al-Ouqaili, M. T. & Al-Meani, . S. A. (2020). Ciprofloxacin- and gentamicin-mediated inhibition of Pseudomonas aeruginosa biofilms is enhanced when combined the volatile oil from Eucalyptus camaldulensis. SRP, 11 (7), 98-105.

2. Methods

2.1. Search strategy and Study selection

The Preferred Reporting Items for Systematic Reviews and Meta-analysis (PRISMA) was followed to conduct this study (16). We used PubMed, Google scholar and Web science as search strategies. Articles were identified using MeSH terms and keywords of the title and by using Boolean operators (OR, AND, NOT) such as Factors OR Bacteria OR (“Gastrointestinal Microbiome/drug effects"[Mesh] OR "Gastrointestinal /etiology"[Mesh] AND Carriage OR Colonization OR ("Asymptomatic Infections/epidemiology"[Mesh] OR "Asymptomatic Infections/mortality"[Mesh] OR "Asymptomatic Infections/nursing"[Mesh] OR "Asymptomatic Infections/therapy"[Mesh]) AND Resistance OR ("Drug Resistance OR Bacterial/drug effects"[Mesh] OR "Drug Resistance OR “Extended-spectrum betalactamase-producing *Enterobacteriaceae* /carbapenem-producing *Enterobacteriaceae*"[Mesh] OR "Drug Resistance OR Bacterial/etiology"[Mesh] AND Inpatient OR Hospitalized ORPatient OR("CrossInfection/complications"[Mesh]OR"CrossInfection/diagnosis"[Mesh]OR"CrossInfection/drugtherapy"[Mesh]OR“CrossInfection/epidemiology"[Mesh]OR"CrossInfection/etiology"[Mesh]. All studies starting from January1, 2016 to August10, 2022 were assessed, extracted and refined. All citations were exported to Endnote software and duplicates were removed.

2.2. Eligibility Criteria.

Selection citeria checklist was developed by the authors before identifying appropriately published relevant full-text articles.

Inclusion: All studies which fulfilled at least the following criteria were incorporated in this systematic review and meta-analysis: Case-control, cohort and cross-sectional studies with human

fecal specimens, hospitalized patients, studies reported ESBL-PE and/ or CRE, with or without MDR of Enterobacteriaceae.

Exclusion: Case reports, retrospective studies, unrelated articles and specimens other than stool or fecal were excluded.

PICOS criteria

Participants: Any hospitalized patients

Intervention: Not applicable

Comparator: Not applicable

Outcome: ESBL-PE, CRE, MDR, *E. coli*, *K. pneumoniae,* odds ratio

Study design: Case-control, cohort and cross-sectional

Study setting: Any setting across the globe

2.3. Data Screening, Extraction, and Management.

Online records from different databases and directory were exported properly to ENDNOTE reference software version 8.2. The records were merged into one folder to identify and remove duplicate articles with the help of ENDNOTE or manual tracing way as there are several possibilities of citation styles per article. Data screening was performed by three reviewers (DA, AA and AAN) who independently screen the titles and abstracts of all relevant articles from literature search databases based on the predefined eligibility criteria.

Data extraction format included principally first author, study design, study setting, publication year, study site in the country, sampling technique, sample size, population characteristics, the age

group of study participants, the prevalence of ESBL-PE, diagnostic methods, specimen types, and types of ESBL-PE isolates. In cases of insufficient/ incomplete data, the authors independently reviewed the full-text of the article for further information and clarification. Authors designed a data extraction form adopted from the Preferred Reporting Items for Systematic Reviews and Meta Analyses (PRISMA), 2020 flow diagram. Finally, a total of 20 eligible articles were included in the study as indicated in (Figure1) (16). PRISMA 2020 check list was used to indicate where eah detail descriptions found as shown in the supplementary file (see S1 2020 check list).

2.4. Study Population.

All age groups, gender (male/female), and any ethnic groups live in different countries in the world were included in this study.

I think not to write in the world but most of the world at which there are studies from the countries which are not cited..

2.5. Outcome.

The main outcomes of interest were the prevalence of ESBL-PE colonization, CRE colonization, MDR and risk factors among hospitalized patients from fecal specimens across the globe. *E. coli*, *K. pneumoniae* and comorbidities were also measured.

2.6. Quality Assessment

Critical appraisal of the studies was performed by assigned reviewers (authors) to ensure the accuracy and consistency of data. The quality of studies was assessed using standard critical appraisal tools prepared by Joanna Briggs Institute (JBI) (17). The purpose of the appraisal was to assess the methodological quality of studies, the possibility of bias in its design and statistical analysis. The JBI appraisal checklist contains 8, 10 and 11 questions for cross-sectional, case-control and cohort studies respectively. Two independent authors (AA and AAN) were assigned to assess the quality of each study and the discrepancies were solved by taking the average score.

Finally, five point (5) was taken as a cut of point, and studies with a score of five and above for "yes" were included in the systematic review and meta-analysis. The total score is calculated by counting the number of "yes" in each row and variables are scored out of 100% and quality score is defined as low if $< 60\%$, medium if 60–80% and high if $> 80\%$. Forty five percent of the studies have high level of quality score as indicated in (Table 1).

Table 1. Quality assessment of the studies included in the systematic review and meta-analysis.

Author	Design	Q1	Q2	Q3	Q4	Q5	Q6	Q7	Q8	Q9	Q10	Q11	Scored	100%	Quality
Desta, *et al*	CS	Yes	Yes	Yes	Yes	No	No	Yes	Yes				6	75	Medium
Baljin, *et al*	C	Yes	Yes	Yes	No	No	No	Yes	Yes	No	Yes	Yes	7	64	Medium
Aklilu, *et al*	CS	No	Yes	Yes	Yes	Yes	Yes	Yes	Yes				7	88	High
Mahamat, *et al*	CC	Yes	Yes	UC	Yes	Yes	No	No	Yes	No	Yes		6	60	Medium
Abdallah, *et al*	CS	Yes	Yes	Yes	Yes	No	No	Yes	Yes				6	75	Medium
Ouédraogo, *et al*	CC	Yes	Yes	Yes	Yes	Yes	No	No	Yes	No	Yes		7	70	Medium
Ebrahimi, *et al*	CS	Yes	Yes	Yes	Yes	Yes	Yes	Yes	Yes				8	100	High
Pilmis , *et al*	CS	Yes	Yes	Yes	Yes	No	No	Yes	Yes				6	75	Medium
Kizilates, *et al*	CS	Yes	Yes	Yes	Yes	No	No	Yes	Yes				6	75	Medium
Markovska, *et al*	CC	Yes	Yes	No	Yes	Yes	Yes	No	No	Yes	UC		6	60	Medium
Kibwana, *et al*	CS	Yes	Yes	Yes	Yes	Yes	Yes	Yes	Yes				8	100	High
Godonou, *et al*	CS	Yes	Yes	Yes	Yes	Yes	Yes	Yes	Yes				8	100	High
Kiddee, *et al*	C	Yes	Yes	Yes	Yes	Yes	No	Yes	Yes	No	Yes	Yes	9	82	High
Babu, *et al*	CC	Yes	Yes	Yes	Yes	Yes	No	No	Yes	Yes	Yes		8	80	Medium
Hamprecht, *et al*	CS	Yes	Yes	Yes	Yes	Yes	Yes	Yes	Yes				8	100	High
Mittal, *et al*	CC	Yes	Yes	UC	Yes	Yes	Yes	Yes	Yes	Yes	Yes		9	90	High
Yan, *et al*	C	Yes	Yes	Yes	No	No	No	Yes	Yes	No	Yes	Yes	7	64	Medium
McConville, *et al*	C	Yes	Yes	Yes	Yes	Yes	No	Yes	Yes	No	Yes	Yes	9	82	High
Tran, *et al*	CS	Yes	Yes	Yes	Yes	Yes	Yes	Yes	Yes				8	100	High
Al Fadhli, *et al*	CS	Yes	Yes	Yes	Yes	No	No	Ye	Yes				6	75	Medium

Legend: CC= Case-control, CS=Cross-sectional, Cohort, UC= Unclear

2.7. Data Synthesis, Analysis, and Reporting

Data were imported from Microsoft Excel 2019 to Stata version16.0MP software for statistical analysis. The pooled estimation of ESBL-EP, CRE, MDR and odds ratio with a 95% confidence interval were performed. Random effect model was used to assess heterogeneity ($I^{2)}$) between studies. I^2 ≤25% was considered as more homogeneity while I^2 ≥ 75% suggested high heterogeneity.

3. Result

3.1 Characteristics of included studies in systematic and meta-analysis

From the available scientific database 342 studies were identified. Of this, 200 duplicate studies were removed. One hundered forty two studies were screened by title and abstract. Profound review was made using their titles, study design, outcomes, and other important parameters using checklists and quality assessment tools. The remaining 34 studies were excluded from the analysis based on specimen source, methodological difference and being related to the included studies. Finally, 20 studies were found to be eligible for full-text evaluation, and that fulfilled the eligibility criteria were included in the final meta- analysis. From the included studies, five of them were case-control studies, four of them were prospective cohort and the others were cross-sectional studies. Regarding their sample size, the smallest sample size was 100 by Abdallah, H.M, *e t al* in 2017 and the largest sample size was 4376 a study by Hamprecht, *et al* in 2016. From the total 20 studies, 15 studies investigated ESBL-PE carriage rate, 5 CRE carriage rate, 5 both ESBL-PE and CRE studies. All populations included in this study were hospitalized patients with different clinical conditions and who stayed more than or equal to 4 days.

Most of the studies used double disk synergy test, Combination disk test and PCR for the confirmation of ESBL and CRE production, however, studies from Ethiopia were not used PCR. In addition, multidrug-resistance (MDR) was assessed. MDR indicates the resistance of bacteria for at least one antibiotic in different class of antibiotics, but six studies did not clearly indicate MDR in their studies. In addition, all studies use rectal swab or stool specimen. Most of the publication year of the studies were in the year of 2016 (30%), 2019 (20%) and 2021(20%) respectively as indicated in (Table 2). Large proportion of studies were included from Asia (44%) followed by Africa (33%) as shown in (Figure 2).

3.2. Laboratory methods used to confirm ESBL and CRE production by *Enterobacteriaceae*.

In this meta-analysis, we observed that most studies used combination disk test (CDT) and double disk synergy test (DDST) while a few studies include E-test and PCR to screen ESBL-PE and CRE producing *Enterobacteriaceae*.

Please to use the following references and refer to the works in the following table and the country. This is important for your study to be more comprehensive:-

1-AL-KUBAISY SH, HUSSEIN, RA, AL-OUQAILI, MTS. (2020). Molecular Screening of Ambler class C and extended spectrum -lactamases in multi-drug resistant Pseudomonas aeruginosa and selected species of Enterobacteriaceae. International Journal of Pharmaceutical Research | Jul - Sep 2020 | Vol 12 | Issue 3.

2-Al-Ouqaili, MTS, Khalaf EA, Al-Kubaisy SH. (2020). DNA Sequence Analysis of BlaVEB Gene Encoding Multi-drug Resistant and Extended-spectrum -lactamases Producer Isolates of Enterobacteriaceae and Pseudomonas aeruginosa. The Open Microbiology Journal, 4: 40-47.

3-Al-Ouqaili, MTS, Al-Taei, SA, Al-Najjar A. Molecular Detection of Medically Important Carbapenemases Genes Expressed by Metallo--lactamase Producer Isolates of Pseudomonas aeruginosa and Klebsiella pneumoniae. Asian Journal of Pharmaceutics • Jul -Sep 2018 (Suppl) • 12 (3) | S991.

4-Khalaf, EA, Al-Ouqaili, MTS. Molecular detection and sequencing of SHV gene encoding for extended-spectrum -lactamases produced by multidrug resistance some of the Gram-negative bacteria. International Journal of Green Pharmacy • Oct-Dec 2018 (Suppl) • 12 (4) | S910-S918.

Table 2. Characteristics of individual studies included in the current systematic and met-analysis

No	Author	Year	Size	Age	Design	Confirmation	Country	ESBLcarriage	ESBL-E. coli	ESBL-K.pc	MDR
1	Desta, *et al* *(18)*	2016	267	All	CS	CDT	Ethiopia	139 (52%)	106(39.7%)	44(16.5%)	99% (150/151)
2	Baljin,*et al* *(19)*	2021	158	All	C	CDT+PCR	Mongolia	110 (69.6%)	87 (55%)	15 (9.5%)	66% (85/128)
3	Aklilu, *et al* *(20)*	2019	421	All	CS	CDT	Ethiopia	146(34.7%)	62 (14.7 %)	60 (14.3)	71% (99/140)
4	Mahamat,*et al* *(21)*	2019	100	All	CC	DDST+PCR	Chad	51 (51%)	35 (35%)	11 (11%)	90% (46/51)
5	Abdallah, *et al* *(22)*	2017	100	All	CS	VITEK+PCR	Egypt	68 (68%)	51 (51%)	19 (19%)	30%(22/73)
6	Ouédraogo,*et al* *(23)*	2017	113	All	CC	DDST+PCR	Burkina	47 (42%)	34 (30.1%)	7 (6.2%)	68% (47/69)
7	Ebrahimi, *et al* *(24)*	2016	4343	All	CS	DDST+PCR	Hungaria	323 (7.4%)	179 (4.1%)	185 (4.3)	
8	Pilmis, *et al* *(25)*	2018	554	≥60	CS	DDST+CDT	French	98 (17.7%)	69 (12.6%)	14 (2.5%)	
9	Kizilates, et al (26)	2021	168	All	CS	Vitek+E+PCR	Turkey	67 (39.8%)	39 (23.2%)	28(16.6%)	
10	Markovska,,*et al* *(27)*	2021	311	All	CC	DDST+PCR	Bulgaria	98 (30.2%)	68 (21.8 %)	20 (6.4%)	
11	Kibwana,*et al* *(28)*	2020	196	≥18	CS	CDT	Tanzania	117 (59.7)	80 (40.8%)	37(18.9%)	89%(117/131)
12	Godonou, *et al* *(29)*	2022	105	All	CS	CDT	Togo	85 (80.9%)	73 (69.5%)	24(22.9%)	84% (85/101)
13	Kiddee, *et al* *(30)*	2019	215	All	C	CDT+PCR	Thailand	134 (62.3%)	108(50.2%)	31(14.4%)	98%(156/160)
14	Babu, *et al* *(31)*	2016	220	≥18	CC	CDT+PCR	India	138 (62.7%)	97(44.1%)	37(16.8%)	50%(61/134)
15	Hamprecht,*et al* *(32)*	2016	4376	≥18	CS	CDT+PCR	German	416 (9.5%)	344 (7.9%)	37 (0.9 %)	
**Colonization rate of CRE**								**Overall CRE**	**CRE-E. coli**	**CREK. pc**	
1	Desta, *et al* *(18)*	2016	267	All	CS	CDT	Ethiopia	5 (2%)	3 (1.1%)	2 (0.8 %)	
2	Mittal, *et al* *(33)*	2016	200	All	CC	DDST+PCR	India	71 (35.5%)	30 (15%)	9 (4.5%)	54 (27/50)
3	Yan, *et al* *(34)*	2020	150	All	C	Vitek +PCR	China	25 (16.6%)	6 (4.0%)	17(11.3%)	96% (25/26)
4	McConville, *et al* *(35)*	2017	338	≥ 18	C	Vitek	Colombia	94 (28%)	39 (11.5%)	42(12.4%)	97% (35/36)
5	Tran, *et al* *(36)*	2019	2233	All	CS	Vitek	Vietnam	1165 (52%)	682(30.5%)	805(36.%)	
6	AlFadhli ,*et al* *(37)*	2020	590	≥18	CS	PCR	Kuwait	58 (9.8)	11 (1.9%)	29 (4.9%)	100% (56/56)
7	Abdallah, *et al* *(22)*	2017	100	All	CS	CDT+PCR	Egypt	5 (5%)		5 (5%)	
8	Kizilates, *et al* *(26)*	2021	168	All	CS	Vitek+E+PCR	Turkey	21 (12.5%)	10(47.6%)	11(52.4%)	
9	Markovska, *etal* *(27)*	2021	311	All	CC	DDST+PCR	Bulgaria	3 (1%)		3 (1%)	
10	Hamprecht,*et al* *(32)*	2016	4376	≥18	CS	CDT+PCR	German	5 (0.11)			

Legend: CC= Case-control, CS= Cross-sectional, C= Cohort, CDT= Combination disk test, DDST= double disk synergy test, E= E-test.

3.3. The pooled estimate of ESBL-PE and CRE Colonization among hospitalized patients

Separate analysis was made for ESBL-PE and CRE colonization. Accordingly, the overall pooled estimate of ESBL-PE was 45.6% (95% CI:34.11-57.10, P< 0.001) with high level of heterogeneity (I^2 =99.63%) as indicated in (Figure 2). The pooled estimate of CRE colonization was 16.19% (95% CI= 5.46-26.91, P <0.001) with high level of heterogeneity (I^2=99.78%) as demonstrate in (Figure 3). The predominant ESBL producer was *Escherichia coli*, 32.99 % (95% CI=23.28-42.69, P<0.001) followed by *K. pneumoniae*, 11% (95% CI=7.98-14.89, P<0.001) as shown in (Figure 4 and 5) respectively. In addition, the most common CRE producer was *K. pneumoniae*,50% (95% CI=25.09-73.96, P<0.001) in (Figure 6), and the least common CRE producer was *E. coli,43%* (95% CI=27.93-58.68, P<0.001) as depicted in (Figure7).

3.4. Meta-analysis of publication bias

Publication bias was assessed to investigate problems related to published and unpublished papers, the analysis showed assymetric funnel plot which indicates the presence of publication bias as shown in (Figure 8).

3.5. Subgroup analysis of ESBL-PE and CRE colonization by region

Sub group analysis of ESBL-PE and CRE colonization was performed by geographical locations. The highest pooled estimate of ESBL-PE was reported in Asian region,58.23% (95% CI=45.79-70.67, P<0.001, I^2 =91.96%) followed by the African region,55.36% (95% CI=43.68-67.03, P<0.001, I^2 = 94.42%,) The least pooled estimate was recorded from Europe region, 15.96% (95% CI=6.01-25.91, P<0.001, I^2 =99.52%,) as indicated in (Figure 9). When stratified by country, the top four EBLE-PE prevalence were reported in Togo 80.90% (95% CI=72.98-88.82), Mongolia 69.60% (95% CI= 62.19-77.01), Egypt 68.0% (95% CI= 58.52-77.48), Thailand 62.30% (95% CI=55.65-68.95) and India 61.0% (95% CI=54.38-67.61) (Figure 9).

Similarly, CRE colonization subgroup analysis was made and relatively higher pooled prevalence was found in Asia than Europe and Africa, 28.43% (95% CI=13.78-43.07, P<0.001, I^2 = 98.77), 4.12% (95% CI=-3.27-11.51, P<0.001, I^2 =98.90) and 2.64% (95% CI= 0.23-5.04, P=0.28, I^2 =15.76) respectively as depicted in (Figure10). High CRE colonization was reported in Vietnam, 52.0%(95%CI=49.90-54.10).

3.6. Pooled estimate of MDR

Only 13 studies were eligible for multi drug resistance (MDR) meta-analysis and the pooled estimate was 76% (95% CI=64.29-88.48, P<0.001) as shown in (Figure11). Most of the studies defined MDR as bacteria resistant to two or three class of antimicrobials**.** The highest and lowest MDR was recorded at 99.3% (95%CI=97-22-101.38) and 45.5% (95%CI=36.82-54.18) respectively.

3.7. Pooled estimate of odds ratio for history of hospitalization

The majority of studies defined previous hospitalizations as admission within the last 3, 6 or 12 months. Five studies analyzed to figure out whether previous hospitalization can be a risk factor or not for fecal colonization with ESBL-producing *Enterobacteriaceae*, and we found a 0.93 pooled estimate odds ratio ((95% CI= 0.54-1.32) as indicated in (Figure12). Five studies were eligible for meta-analysis to assess the impact of prolonged hospitalization for ESBL colonization. These studies defined hospital stay as patients admitted for ≥ 7 days, and the analysis reported that the odds of 0.94 (95% CI, 0.52-1.37) as indicated in (Figure 13).

3.8. Pooled estimate of odds ratio for history of antibiotic use

History of antibiotic treatment was analyzed in seven studies, and a history of antibiotic treatment in the last 3 and 6 months was found to be a risk factor for ESBL-PE colonization. The pooled

estimate was 1.45 (95% CI= 0.81-2.08) as shown in (Figure 14). In addition, current antibiotic use during admission such as carbapenem and fluoroquinolone was assessed among two studies. Carbapenem treatment found to be associated with the colonization of CRE (OR:1.72, 95% CI:1.05-2.40) while fluoroquinolone (OR:0.98, 95% CI:0.59-1.38) was not associated as indicated in (Figure 15 and 16).

3.9. Meta-analysis of the association between comorbidities and ESBL-PE or CRE colonization.

As indicated in the supplementary file (Table3), meta-analysis was performed to assess the association of comorbidities with ESBL-PE or CRE colonization. Accordingly, two studies assessed for any chronic diseases and showed an increased risk for ESBL colonization, 2.02 (95% CI= 1.17-2.87). Related to renal diseases, three studies were analyzed and associated with CRE colonization with the odds of 1.78 (95% CI=0.06-3.50), and another two studies with diabetics showed that CRE colonization can be a risk factor with 1.42 (95% CI=0.20-2.65). In addition, two cohort studies were separately analyzed to assess the CRE colonization rate at the time of admission and after ≥7 days of admission. The result showed that there was an increased rate of CRE colonization at admission and after admission with odds of 14.77 (95% CI= -1.35-30.90) and 45.63 (95% CI=-0.86-92.12) respectively.

Table 3. Meta-analysis of association between comorbidities and ESBL-PE /CRE colonization.

Comorbidities	Included studies	Heterogeneity (I^2)	P-value	OR	95% CI
Chronic diseases	2	0.00%	<0.001	2.02	1.17-2.87
Surgery	2	0.00%	0.01	0.70	0.21-1.18
Renal disease	3	49.56%	0.04	1.78	0.06-3.50
Diabetics	2	0.00%	0.02	1.42	0.20-2.65
Any catheterization	3	0.00%	<0.001	1.04	0.99-1.09
At admission	2	99.54%	<0.001	14.77	-1.35-30.90
After admission	2	99.90%	<0.001	45.63	0.86-92.12

Legends: OR= Odds ratio, CI= Confidence interval

4. Discussion

Our systematic review and meta-analysis assessed factors influencing colonization with MDRB in different studies across the globe. We analyzed pooled estimate of MDR, ESBL-PE and CRE colonization, comorbidities, and risk factors of MDR colonization reported from January1,2016-August 10, 2022. It also focused on *E. coli* and *K. pneumoniae* because these bacteria are identified as MDR bacteria that are strongly associated with nosocomial infection (38). In this study, the source of specimen was stool /rectal swab among hospitalized patients (ICU or general ward patients).

The emergence of multidrug-resistant bacteria has become a global priority concern (39). Particularly, ESBL-PE and CRE are highly linked to prolonged hospitalization, morbidity, mortality and extra treatment cost (40, 41). In the present study, a meta-analysis compiled from different countries revealed a pooled estimate of ESBL-PE colonization among hospitalized patients, 45.6% (95% CI: 34.11-57.10) with the lowest prevalence in Hungary,7.4% and the highest reported in Togo, 80.9% (24, 29) respectively. The highest report in Togo could be due to its' recent publication period, which was in 2022. Our finding was higher than from a systematic study of sub-Saharan Africa on admitted patients with a pooled prevalence of 32% (42). This variation might be due to geographical location, our study focused on three continents (Africa, Asia and Europe) which could increase the prevalence of ESBL-PE colonization compared to the sub-Saharan Africa study. Similarly, lower result was also reported from another systematic review, 18% (43) that was mainly from European countries conducted before 2016. This gap could be due to study period and geographical location.

It is important to refer to the following reference:
Al-Ouqaili, MTS., Jal'oot, AS., Badawy, AS. Identification of an OprD and bla(IMP) Gene-mediated Carbapenem Resistance in Acinetobacter baumannii and Pseudomonas aeruginosa among Patients with Wound Infections in Iraq. Asian Journal of Pharmaceutics., volume 12, issue 3, 2019 Page S959-S965-

In this meta-analysis, the predominant ESBL-PE were *E. coli* and *K. pneumoniae* with pooled estimate of 32.99 % and 11% respectively. These figures were much lower than studies in Togo

(69.5% vs22.9%), India (56% vs 43%), and Ethiopia (54.9% vs 33.5%) (29, 44, 45) respectively. Similarly, a large prospective study showed high prevalence of *E. coli*,57% and *K. pneumoniae*,43% (46). However, *K. pneumoniae* was reported as a dominant ESBL-PE with a prevalence of 51.7% over *E. coli*,46.4% (47). This inconsistent finding might be related with study setting variations and detection methods. In the current meta-analysis, the pooled estimate of CRE colonization was 16.19% (95% CI: 5.46-26.91), which was much greater than a previous systematic review and meta-analysis study that reported CRE colonization at 3.0% mainly from America and Europe (48). This greater prevalence in our meta-analysis may be due to geographical locations, most of included studies were from African and Asian regions. Another possible reason might be due to number of studies included, we included 10 studies with CRE colonization while they included 4 studies. In contrast, our finding was lower than studies in Thailand, 69.7% (49), India, 18% (50) and Egypt,28% (51). This disparity could be due to our result was an average from different countries, and another reason may be related with the presence of serious comorbidities, medical intervention and overuse of antibiotics in their study. From the total pooled carbapenem resistant *Enterobacteriaceae, K. pneumoniae* accounted 50% and *E. coli* 43%. This was inconsistent with study from India, *E.coli* 71.4%, *K. pneumoniae* 23.8% (52), and another report from Mexico showed *E.coli* 72.7% and *K. pneumoniae* 23.6% (53). This variation might be due to the distribution of bacteria changes over time across countries.

In this study, subgroup meta-analysis was performed which showed the highest pooled prevalence of ESBL-PE colonization in Asia, 58.23% (95% CI=45.79-70.67). This high prevalence might be related with high consumption of beta lactam and carbapenem antibiotic and most CTX-M genes were emerged from this continent and spread to other regions (54). Another suggested reason was low control mechanism in antibiotic usage which causes overuse and misuse of antibiotics in the

healthcare facilities and animal husbandry, and considered as highly vulnerable region for antimicrobial resistance (55, 56). Africa was the second most common region that reported significant ESBL-PE colonization rate,55.36% (95% CI=43.68-67.03). The reason for this considerable report might be due to lack of stewardship and limited resources to prevent and control the spread of multidrug-resistance (57). The least ESBL colonization was recorded in Europe regions 15.9%, which was similar to a systematic review and meta-analysis with a prevalence of 15.0% (58). This low prevalence could be explained by the fact that these regions are high income countries that have good infrastructure and steward ship program to prevent and control the spread of multidrug-resistance (57). It might be also related with large sample size; estimates from large population size may lower the pooled estimate.

When we compared at country level in each continent, the highest ESBL-PE colonization was reported in Mongolia, 69.60% (95% CI= 62.19-77.01), Thailand 62.30% (95% CI=55.65-68.95) and India 61.0% (95% CI=54.38-67.61). This high prevalence may be related with study design, for instance Mongolian study was a cohort study which showed an increased colonization rate of ESBL-PE during their 14-day hospitalization while data were taken at the time of admission in the case of Thailand and Indian study. It could be also due to study population and study settings; the Mongolian study used all age groups at trauma center whereas in Thailand and India the study population were above the age of 18 years old.

In African countries, the highest ESBL-PE colonization were reported from Togo, 80.90% (95% CI=72.98-88.82) and Egypt, 68.0% (95% CI= 58.52-77.48). The possible reason for this difference might be due to publication period, example the data from Togo was very recent,2022 which strengthen the increasing trend of antimicrobial resistance globally (59). Study population might

be also considered as a reason for this discrepancy, for example the study in Togo used all admitted patients in all wards whereas in Egypt only gastrointestinal complaints were participated.

In the present systematic review and meta-analysis, CRE colonization subgroup analysis was performed and revealed the highest pooled estimate in Asia region, 28.43% (95% CI=13.78-43.07). Lower colonization rate of CRE was reported in Africa and Europe regions than Asian region, this might be due to detection of CRE colonization was not their target while the Asian regions studied exclusively CRE carriage rate. When stratified by country, higher CRE colonization was reported in Vietnam, 52.0% (95%CI=49.90-54.10) than other Asian countries. This high burden of CRE could be due to large sample size from 12 hospitals that can enhance the probability of detecting more CRE colonization. Additionally, it might be due to over use of carbapenem antibiotics in the community because of lack of control mechanisms in the usage of antibiotics.

Multidrug resistant bacteria are more prevalent in hospitalized patients and cause more infections because of invasive procedures, chronic diseases and over use of antibiotics (60). The rate of multidrug-resistant bacteria range from 30-50 % in china (61) and 89.5% in Africa (62). Nearly 20% of hospital acquired infections are due to multidrug resistant bacteria (63). In our meta-analysis, the pooled prevalence of MDR was 76% (95% CI =64.29-88.48), which was slightly higher than a systematic and meta-analysis in Ethiopia,71% (15). This variation may be related with geographical location, high consumption rate of antibiotics and presence of sever underlining comorbidities in our study population. Another systematic review and meta-analysis from Asia reported a lower MDR prevalence 66% (64) than our finding. This difference might be due to specific study population that were with diarrheagenic patients and focused on only *E. coli* in their study. On the other hand, our finding was less than a systematic review and meta-analysis reported

from Ethiopia with pooled prevalence of 82.7% (65) and a single study in Debre Brehan, Ethiopia 87.8% (45). This variation could be due to specimen source; we used fecal specimen while they included multiple sample sources. Furthermore, misuse and overuse of antibiotics including poor implementation of antimicrobial stewardship in their study settings can contribute for the increasing rate of MDR.

Several studies identified many risk factors of multidrug-resistant bacteria colonization among hospitalized patients. Such as previous hospitalization, previous antibiotic treatment, length of hospital stays, and co-morbidities like surgery, liver cirrhosis, diabetics, and renal diseases (66-68). In healthcare facilities, intestinal carriage of ESBL-PE is one of the main reservoirs of multidrug-resistant bacteria which have been associated with high risk for developing infections (69, 70). In our meta-analysis, six studies were eligible to assess the association of previous hospitalization and antibiotic treatment with ESBL-PE colonization. The meta-analysis showed that there was no association between previous hospitalization and ESBL colonization, this lack of association might be due to the low number of studies included in the analysis. This finding disagreed with a systematic and meta-analysis study (71), and another individual study (72). However little association was noticed between previous antibiotic treatment and ESBL colonization, which was consistent to a previous study (71). This association might be due to the fact that antibiotic use has been strongly implicated in the development of antimicrobial resistance (73).

Meta-analysis was also performed to assess the association of comorbidities with the acquisition of ESBL-PE or CRE. Chronic diseases have showed an increased risk for ESBL-PE colonization, 2.02 (95% CI, 1.17-2.87). In addition, renal diseases and diabetics were associated with CRE colonization with the odds of 1.78 (95% CI,0.06-3.50) and 1.42 (95% CI, 0.20-2.65) respectively.

This finding was congruent with previous studies in Ethiopia (20, 45), Algeria (74) and India (75). This association may be due to the fact that immunocompromised patients are more prone to be colonized with MDRB. In the current study, colonization of CRE is increased during hospital stay with the odds of 14.77 (95% CI: -1.35-30.90) at the time of admission and 45.63 (95% CI: -0.86-92.12) after ≥7 days of hospital stay, this was consistent with previous studies (19, 66).

5.Limitation of the study

We used only three data bases, this can limit to access full information about published articles. Risk factor estimation was based on limited number of studies, due to inconsistent reports. In addition, articles published other than English were not included.

6.Conclusion

The overall colonization rate of ESBL-PE and CRE were high. Risk factors such as previous antibiotic treatment and hospital stay were associated with ESBL-PE and CRE colonization. In addition, comorbidities such as renal diseases and diabetic diseases were linked with the colonization of ESBL-PE and/or CRE.

Please to add to line 388 as follow: -
antibiotic treatment and the duration in addition to long stay in the hospital

Acknowledgement

We acknowledge the authors of each study. We wish to thank Penn IIZD International Mentoring Initiative for networking opportunities.

Funding

The authors received no specific funding for this work.

Competing interests

K.C. has received research support and/or consulting fees from Pfizer, Takeda, Pacific Biosciences, Vedanta, Genentech, and Abbvie; and is an inventor on U.S. patent 10,722,600 and provisional patent 62/935,035 and 63/157,225.

Availability of data materials

Data is available without restriction

Author contribution

Conceptualization: Dessie Abera, Abel Abera Negash, Kenn CadWell

Data curation: Dessie Abera, Ayinalem Alemu

Formal analysis: Dessie Abera, Ayinalem Alemu

Investigation: Dessie Abera, Ayinalem Alemu, Abel Abera Negash

Methodology: Dessie Abera, Ayinalem Alemu

Supervision: Kenn CadWell, Adane Mihret, Woldearegay Erku Abegaz, Abel Abera Negash

Validation: Woldearegay Erku Abegaz, Abel Abera Negash, Adane Mihret

Writing – original draft: Dessie Abera

Writing – review & editing: Dessie Abera, Ayinalem Alemu, Kenn CadWell, Adane Mihret, Woldearegay Erku Abegaz, Abel Abera Negash. All authors read and approved the final manuscript.

7. References

1. Kim YS, Ho SB. Intestinal goblet cells and mucins in health and disease: recent insights and progress. *Curr Gastroenterol Rep*. 2010;12(5):319-30.

2. Royden A, Ormandy E, Pinchbeck G, Pascoe B, Hitchings MD, Sheppard SK, et al. Prevalence of faecal carriage of extended- spectrum β- lactamase (ESBL)- producing Escherichia coli in veterinary hospital staff and students. *Vet Rec Open*. 2019;6(1):e000307.

3. Chemaly RF, Simmons S, Dale Jr C, Ghantoji SS, Rodriguez M, Gubb J, et al. The role of the healthcare environment in the spread of multidrug-resistant organisms: update on current best practices for containment. *Advances infect Dis*. 2014;2(3-4):79-90.

4. Vincent J-L. Nosocomial infections in adult intensive-care units. *Lancet*. 2003;361(9374):2068-77.

5. Bush K, Bradford PA. Epidemiology of β-lactamase-producing pathogens. Clinical microbiology reviews. 2020;33(2):e00047-19.

6. Peirano G, Pitout JD. Extended-spectrum β-lactamase-producing Enterobacteriaceae: update on molecular epidemiology and treatment options. Drugs. 2019;79(14):1529-41.

7. Peterson E, Kaur P. Antibiotic resistance mechanisms in bacteria: relationships between resistance determinants of antibiotic producers, environmental bacteria, and clinical pathogens. Frontiers in microbiology. 2018;9:2928.

8. Ruppe E, Pitsch A, Tubach F, De Lastours V, Chau F, Pasquet B, et al. Clinical predictive values of extended-spectrum beta-lactamase carriage in patients admitted to medical wards. *Eur J Clin Microbiol Infect Dis*. 2012;31(3):319-25.

9. Organization WH. Antimicrobial stewardship programmes in health-care facilities in low-and middle-income countries: a WHO practical toolkit. 2019.

10. Joubert D, Cullati S, Briot P, Righi L, Grauser D, Ourahmoune A, et al. How to improve hospital admission screening for patients at risk of multidrug-resistant organism carriage: a before-and-after interventional study and cost-effectiveness analysis. BMJ open quality. 2022;11(2):e001699.

11. Strich JR, Palmore TN. Preventing transmission of multidrug-resistant pathogens in the intensive care unit. Infectious Disease Clinics. 2017;31(3):535-50.

12. Fulchini R, Albrich W, Kronenberg A, Egli A, Kahlert C, Schlegel M, et al. Antibiotic-resistant pathogens in different patient settings and identification of surveillance gaps in Switzerland–a systematic review. Epidemiology & Infection. 2019;147.

13. Kiros T, Workineh L, Tiruneh T, Eyayu T, Damtie S, Belete D. Prevalence of Extended-Spectrum β-Lactamase-Producing Enterobacteriaceae in Ethiopia: A Systematic Review and Meta-Analysis. International journal of microbiology. 2021;2021.

14. Chen Y, Chen X, Liang Z, Fan S, Gao X, Jia H, et al. Epidemiology and prediction of multidrug-resistant bacteria based on hospital level. Journal of Global Antimicrobial Resistance. 2022;29:155-62.

15. Beyene AM, Gezachew M, Mengesha D, Yousef A, Gelaw B. Prevalence and drug resistance patterns of Gram-negative enteric bacterial pathogens from diarrheic patients in Ethiopia: A systematic review and meta-analysis. PloS one. 2022;17(3):e0265271.

16. Haddaway NR, Page MJ, Pritchard CC, McGuinness LA. PRISMA2020: An R package and Shiny app for producing PRISMA 2020- compliant flow diagrams, with interactivity for optimised digital transparency and Open Synthesis. Campbell Systematic Reviews. 2022;18(2):e1230.

17. Porritt K, Gomersall J, Lockwood C. JBI's systematic reviews: study selection and critical appraisal. AJN The American Journal of Nursing. 2014;114(6):47-52.

18. Desta K, Woldeamanuel Y, Azazh A, Mohammod H, Desalegn D, Shimelis D, et al. High gastrointestinal colonization rate with extended-spectrum β-lactamase-producing Enterobacteriaceae in hospitalized patients: emergence of carbapenemase-producing K. pneumoniae in Ethiopia. *PloS One*. 2016;11(8):e0161685.

19. Baljin B, Gurjav U, Tulgaa K, Baldan G, Gunchin B, Sandag T, et al. High Acquisition Rate of Gram-Negative Multi-Drug Resistant Organism Colonization During Hospitalization: A Perspective from a High Endemic Setting. Infection and Drug Resistance. 2021;14:3919.

20. Aklilu A, Manilal A, Ameya G, Woldemariam M, Siraj M. Gastrointestinal Tract Colonization Rate of Extended-Spectrum Beta-Lactamase-and Carbapenemase-Producing Enterobacteriaceae and Associated Factors Among Hospitalized Patients in Arba Minch General Hospital, Arba Minch, Ethiopia. Infection and Drug Resistance. 2020;13:1517.

21. Ouchar Mahamat O, Tidjani A, Lounnas M, Hide M, Benavides J, Somasse C, et al. Fecal carriage of extended-spectrum β-lactamase-producing Enterobacteriaceae in hospital and community settings in Chad. *Antimicrobial Resistance & Infection Control*. 2019;8(1):1-7.

22. Abdallah H, Alnaiemi N, Reuland E, Wintermans B, Koek A, Abdelwahab A, et al. Fecal carriage of extended-spectrum β-lactamase-and carbapenemase-producing Enterobacteriaceae in Egyptian patients with community-onset gastrointestinal complaints: a hospital-based cross-sectional study. *Antimicrobial Resistance & Infection Control*. 2017;6(1):1-7.

23. Ouédraogo A-S, Sanou S, Kissou A, Poda A, Aberkane S, Bouzinbi N, et al. Fecal carriage of Enterobacteriaceae producing extended-spectrum beta-lactamases in hospitalized patients and healthy community volunteers in Burkina Faso. *Microbial Drug Resistance*. 2017;23(1):63-70.

24. Ebrahimi F, Mózes J, Monostori J, Gorácz O, Fésűs A, Majoros L, et al. Comparison of rates of fecal colonization with extended- spectrum beta- lactamase- producing enterobacteria among patients in different wards, outpatients and medical students. *Microbiol Immunol*. 2016;60(5):285-94.

25. Pilmis B, Cattoir V, Lecointe D, Limelette A, Grall I, Mizrahi A, et al. Carriage of ESBL-producing Enterobacteriaceae in French hospitals: the PORTABLSE study. *Journal of Hospital Infection*. 2018;98(3):247-52.

26. Kizilates F, Yakupogullari Y, Berk H, Oztoprak N, Otlu B. Risk factors for fecal carriage of extended-spectrum beta-lactamase-producing and carbapenem-resistant Escherichia coli and Klebsiella pneumoniae strains among patients at hospital admission. *Am J Infect Control* 2021;49(3):333-9.

27. Markovska R, Stankova P, Stoeva T, Ivanova D, Pencheva D, Kaneva R, et al. Fecal Carriage and Epidemiology of Extended-Spectrum Beta-Lactamase/Carbapenemases Producing Enterobacterales Isolates in Bulgarian Hospitals. *Antibiotics*. 2021;10(6):747.

28. Kibwana UO, Majigo M, Kamori D, Manyahi J. High fecal carriage of extended Beta Lactamase producing Enterobacteriaceae among adult patients admitted in referral hospitals in Dar es Salaam, Tanzania. *BMC Infect Dis*. 2020;20(1):1-7.

29. Godonou A, Lack F, Gbeasor-Komlanvi F, Konlani L, Dossim S, Ameyapoh Y, et al. High faecal carriage of extended-spectrum beta-lactamase producing Enterobacteriaceae (ESBL-PE) among hospitalized patients at Sylvanus Olympio Teaching Hospital, Lomé, Togo in 2019. *Afr J Clin Exper Microbiol*. 2021;23(1):40-8.

30. Kiddee A, Assawatheptawee K, Na-Udom A, Boonsawang P, Treebupachatsakul P, Walsh TR, et al. Risk factors for extended-spectrum β-lactamase-producing Enterobacteriaceae carriage

in patients admitted to intensive care unit in a tertiary care hospital in Thailand. *Microbial Drug Resistance*. 2019;25(8):1182-90.

31. Babu R, Kumar A, Karim S, Warrier S, Nair SG, Singh SK, et al. Faecal carriage rate of extended-spectrum β-lactamase-producing Enterobacteriaceae in hospitalised patients and healthy asymptomatic individuals coming for health check-up. *Journal of global antimicrobial resistance*. 2016;6:150-3.

32. Hamprecht A, Rohde AM, Behnke M, Feihl S, Gastmeier P, Gebhardt F, et al. Colonization with third-generation cephalosporin-resistant Enterobacteriaceae on hospital admission: prevalence and risk factors. *Journal of Antimicrobial Chemotherap*y. 2016;71(10):2957-63.

33. Mittal G, Gaind R, Kumar D, Kaushik G, Gupta KB, Verma P, et al. Risk factors for fecal carriage of carbapenemase producing Enterobacteriaceae among intensive care unit patients from a tertiary care center in India. *BMC Microbiol*. 2016;16(1):1-10.

34. Yan L, Sun J, Xu X, Huang S. Epidemiology and risk factors of rectal colonization of carbapenemase-producing Enterobacteriaceae among high-risk patients from ICU and HSCT wards in a university hospital. *Antimicrobial Resistance & Infection Control*. 2020;9(1):1-8.

35. McConville TH, Sullivan SB, Gomez-Simmonds A, Whittier S, Uhlemann A-C. Carbapenem-resistant Enterobacteriaceae colonization (CRE) and subsequent risk of infection and 90-day mortality in critically ill patients, an observational study. *PloS One*. 2017;12(10):e0186195.

36. Tran DM, Larsson M, Olson L, Hoang NT, Le NK, Khu DT, et al. High prevalence of colonisation with carbapenem-resistant Enterobacteriaceae among patients admitted to Vietnamese hospitals: risk factors and burden of disease. *Journal of Infection*. 2019;79(2):115-22.

37. Al Fadhli AH, Jamal WY, Rotimi VO. Prevalence of carbapenem-resistant Enterobacteriaceae and emergence of high rectal colonization rates of bla OXA-181-positive isolates in patients admitted to two major hospital intensive care units in Kuwait. *PloS One*. 2020;15(11):e0241971.

38. Ponyon J, Kerdsin A, Preeprem T, Ungcharoen R. Risk Factors of Infections Due to Multidrug-Resistant Gram-Negative Bacteria in a Community Hospital in Rural Thailand. Tropical Medicine and Infectious Disease. 2022;7(11):328.

39. Blair J, Webber MA, Baylay AJ, Ogbolu DO, Piddock LJ. Molecular mechanisms of antibiotic resistance. *Nat Rev Microbiol*. 2015;13(1):42-51.

40. Hansen GT. Continuous evolution: perspective on the epidemiology of carbapenemase resistance among enterobacterales and other gram-negative bacteria. *Infect Dis Ther*. 2021;10(1):75-92.

41. Organization WH. Antimicrobial resistance global report on surveillance: 2014 summary. World Health Organization, Geneva, Switzerland, 2014.; 2014.

42. Lewis JM, Lester R, Garner P, Feasey NA. Gut mucosal colonisation with extended-spectrum beta-lactamase producing Enterobacteriaceae in sub-Saharan Africa: a systematic review and meta-analysis. *Wellcome open research*. 2019;4.

43. Flokas ME, Alevizakos M, Shehadeh F, Andreatos N, Mylonakis E. ESBL-producing Enterobacteriaceae Colonization in Long-Term Care Facilities (LTCFs): A Systematic Review and Metaâ analysis. International Journal of Antimicrobial Agents. 2017.

44. Mulki SS, Ramamurthy K, Bhat S. Fecal carriage of extended-spectrum beta-lactamase-producing Enterobacteriaceae in intensive care unit patients. *Indian J Crit Care Med*. 2017;21(8):525.

45. Shenkute D, Legese MH, Yitayew B, Mitiku A, Engidaye G, Gebremichael S, et al. High Magnitude of Fecal Carriage of Extended-Spectrum Beta-Lactamase-Producing Enterobacteriaceae at Debre Berhan Comprehensive Specialized Hospital, Ethiopia. Infection and Drug Resistance. 2022;15:2445.

46. Razazi K, Mekontso Dessap A, Carteaux G, Jansen C, Decousser J-W, de Prost N, et al. Frequency, associated factors and outcome of multi-drug-resistant intensive care unit-acquired pneumonia among patients colonized with extended-spectrum β-lactamase-producing Enterobacteriaceae. Annals of intensive care. 2017;7(1):1-7.

47. Sadek M, Halim M, Fouda AM. Detection of blaTEM, blaSHV, and blaCTX-M genes among the Extended-Spectrum β-Lactamases (ESβLs) producing Enterobacteriaceae isolated from hospital-acquired infections and community in Egypt. Al-Azhar International Medical Journal. 2021;2(3):7-14.

48. Soontaros S, Leelakanok N. Association between carbapenem-resistant Enterobacteriaceae and death: A systematic review and meta-analysis. American journal of infection control. 2019;47(10):1200-12.

49. Wangchinda W, Laohasakprasit K, Lerdlamyong K, Thamlikitkul V. Epidemiology of Carbapenem-Resistant Enterobacterales Infection and Colonization in Hospitalized Patients at a University Hospital in Thailand. Infection and Drug Resistance. 2022;15:2199-210.

50. Mohan B, Prasad A, Kaur H, Hallur V, Gautam N, Taneja N. Fecal carriage of carbapenem-resistant Enterobacteriaceae and risk factor analysis in hospitalised patients: a single centre study from India. *Indian J Med Microbiol*. 2017;35(4):555-62.

51. El-Defrawy I, Gamal D, El-Gharbawy R, El-Seidi E, El-Dabaa E, Eissa S. Detection of intestinal colonization by carbapenem-resistant Enterobacteriaceae (CRE) among patients

admitted to a tertiary care hospital in Egypt. *Egyptian Journal of Medical Human Genetics*. 2022;23(1):1-8.

52. Ravikant M, Felix J. Prevalence and Risk Factor Analysis of Faecal Carriage of Carbapenem Resistant Enterobacteriaceae in a Tertiary Care Hospital. *Int J Clin Diagn Res*.

53. Torres-Gonzalez P, Cervera-Hernandez ME, Niembro-Ortega MD, Leal-Vega F, Cruz-Hervert LP, García-García L, et al. Factors associated to prevalence and incidence of carbapenem-resistant Enterobacteriaceae fecal carriage: a cohort study in a Mexican tertiary care hospital. *PloS One*. 2015;10(10):e0139883.

54. Hawkey P. Multidrug-resistant Gram-negative bacteria: a product of globalization. Journal of Hospital Infection. 2015;89(4):241-7.

55. Song J. Antimicrobial resistance control in Asia. AMR control. 2015:41-5.

56. Yam ELY, Hsu LY, Yap EP-H, Yeo TW, Lee V, Schlundt J, et al. Antimicrobial Resistance in the Asia Pacific region: a meeting report. BioMed Central; 2019.

57. Organization WH. Worldwide country situation analysis: response to antimicrobial resistance: summary. Worldwide country situation analysis: response to antimicrobial resistance: summary2015.

58. Alevizakos M, Karanika S, Detsis M, Mylonakis E. Colonisation with extended-spectrum β-lactamase-producing Enterobacteriaceae and risk for infection among patients with solid or haematological malignancy: a systematic review and meta-analysis. International journal of antimicrobial agents. 2016;48(6):647-54.

59. Murray CJ, Ikuta KS, Sharara F, Swetschinski L, Aguilar GR, Gray A, et al. Global burden of bacterial antimicrobial resistance in 2019: a systematic analysis. The Lancet. 2022;399(10325):629-55.

60. Van Duin D, Paterson DL. Multidrug-resistant bacteria in the community: trends and lessons learned. *Infect Dis Clin*. 2016;30(2):377-90.

61. Chen Q, Li D, Beiersmann C, Neuhann F, Moazen B, Lu G, et al. Risk factors for antibiotic resistance development in healthcare settings in China: a systematic review. Epidemiology & Infection. 2021:1-30.

62. Agyepong N, Govinden U, Owusu-Ofori A, Essack SY. Multidrug-resistant gram-negative bacterial infections in a teaching hospital in Ghana. Antimicrobial Resistance & Infection Control. 2018;7(1):1-8.

63. Sievert DM, Ricks P, Edwards JR, Schneider A, Patel J, Srinivasan A, et al. Antimicrobial-resistant pathogens associated with healthcare-associated infections summary of data reported to the National Healthcare Safety Network at the Centers for Disease Control and Prevention, 2009–2010. *Infection Control & Hospital Epidemiology*. 2013;34(1):1-14.

64. Salleh MZ, Nik Zuraina NMN, Hajissa K, Ilias MI, Deris ZZ. Prevalence of Multidrug-Resistant Diarrheagenic Escherichia coli in Asia: A Systematic Review and Meta-Analysis. Antibiotics. 2022;11(10):1333.

65. Abayneh M, Worku T. Prevalence of multidrug-resistant and extended-spectrum beta-lactamase (ESBL)-producing gram-negative bacilli: a meta-analysis report in Ethiopia. Drug Target Insights. 2020;14:16.

66. Prado V, Hernández-Tejero M, Mücke MM, Marco F, Gu W, Amoros A, et al. Rectal colonization by resistant bacteria increases the risk of infection by the colonizing strain in critically ill patients with cirrhosis. Journal of Hepatology. 2022;76(5):1079-89.

67. Zaha DC, Kiss R, Hegedűs C, Gesztelyi R, Bombicz M, Muresan M, et al. Recent advances in investigation, prevention, and management of healthcare-associated infections (HAIs): resistant

multidrug strain colonization and its risk factors in an intensive care unit of a University Hospital. BioMed research international. 2019;2019.

68. Lin I-W, Huang C-Y, Pan S-C, Chen Y-C, Li C-M. Duration of colonization with and risk factors for prolonged carriage of multidrug resistant organisms among residents in long-term care facilities. Antimicrobial Resistance & Infection Control. 2017;6(1):1-7.

69. Oussayeh I, Moussaid F, Traoré AO, Touiti A, Elkhayari M, Soraa N, et al. Epidemiology, risk factors and outcomes of multidrug-resistant bacteria colonization in a Moroccan Medical Intensive Care Unit. PAMJ-Clinical Medicine. 2021;5(33).

70. Andriatahina T, Randrianirina F, Hariniana ER, Talarmin A, Raobijaona H, Buisson Y, et al. High prevalence of fecal carriage of extended-spectrum β-lactamase-producing Escherichia coli and Klebsiella pneumoniae in a pediatric unit in Madagascar. BMC infectious diseases. 2010;10(1):1-8.

71. Chen G, Xu K, Sun F, Sun Y, Kong Z, Fang B. Risk factors of multidrug-resistant bacteria in lower respiratory tract infections: a systematic review and meta-analysis. Canadian Journal of Infectious Diseases and Medical Microbiology. 2020;2020.

72. Zhu W-m, Yuan Z, Zhou H-y. Risk factors for carbapenem-resistant Klebsiella pneumoniae infection relative to two types of control patients: a systematic review and meta-analysis. Antimicrobial Resistance & Infection Control. 2020;9(1):1-13.

73. Sandiumenge A, Diaz E, Rodriguez A, Vidaur L, Canadell L, Olona M, et al. Impact of diversity of antibiotic use on the development of antimicrobial resistance. Journal of Antimicrobial Chemotherapy. 2006;57(6):1197-204.

74. Medboua-Benbalagh C, Touati A, Kermas R, Gharout-Sait A, Brasme L, Mezhoud H, et al. Fecal carriage of extended-spectrum β-lactamase-producing Enterobacteriaceae strains is

associated with worse outcome in patients hospitalized in the pediatric oncology unit of Beni-Messous hospital in Algiers, Algeria. Microbial Drug Resistance. 2017;23(6):757-63.

75. Saxena S, Kumar S, Kumar V, Ravinder K. Risk Factors Associated with Colonization of Intestine by Carbapenem Resistant Enterobacteriaceae in a Pediatric ICU. Journal of Microbiology and Infectious Diseases. 2020;10(02):75-81.

PRISMA FLOW CHART **2020 (16).**

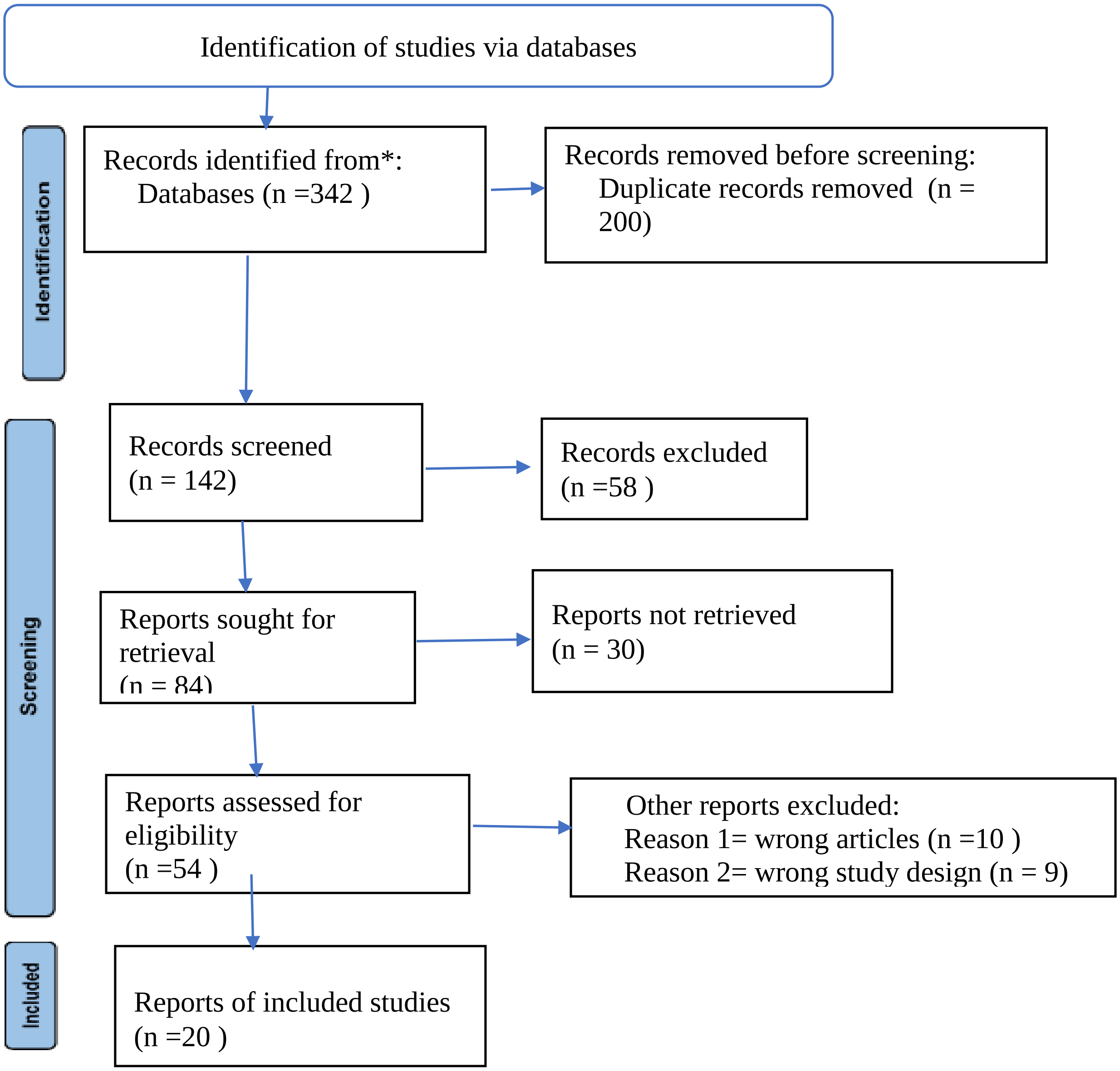

Figure 1. PRISMA FLOW CHART

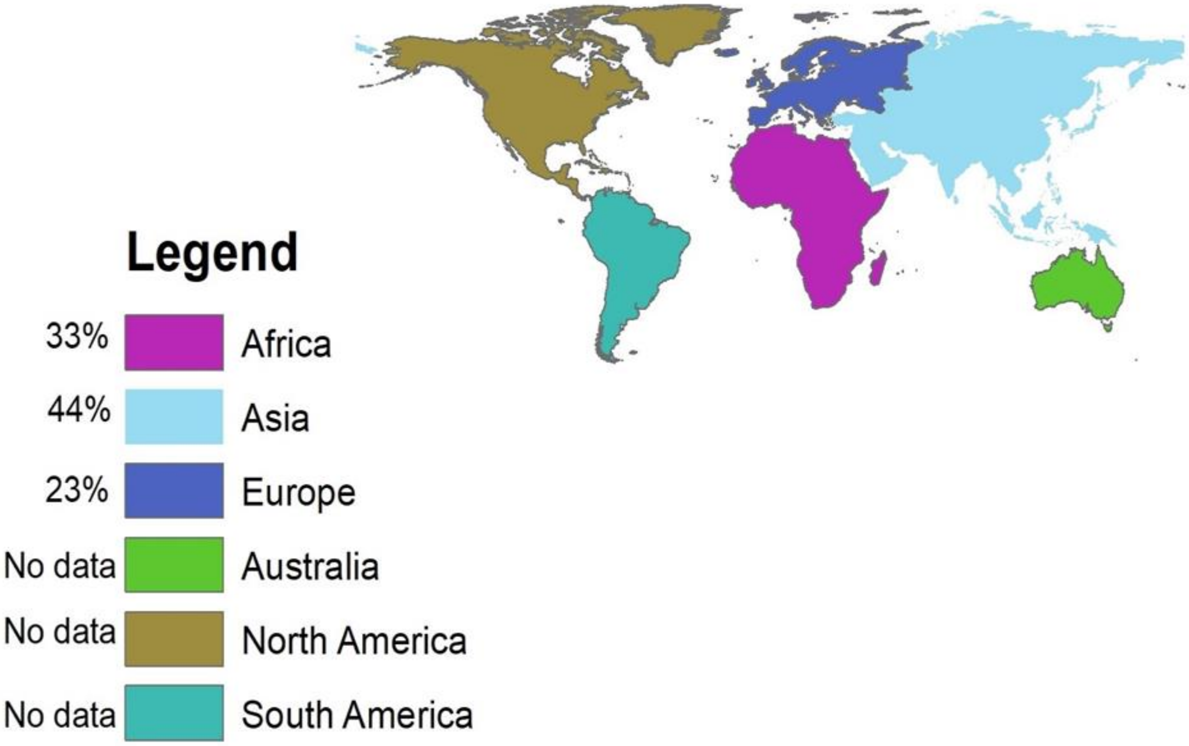

Figure 2. Global regions included in this study

658

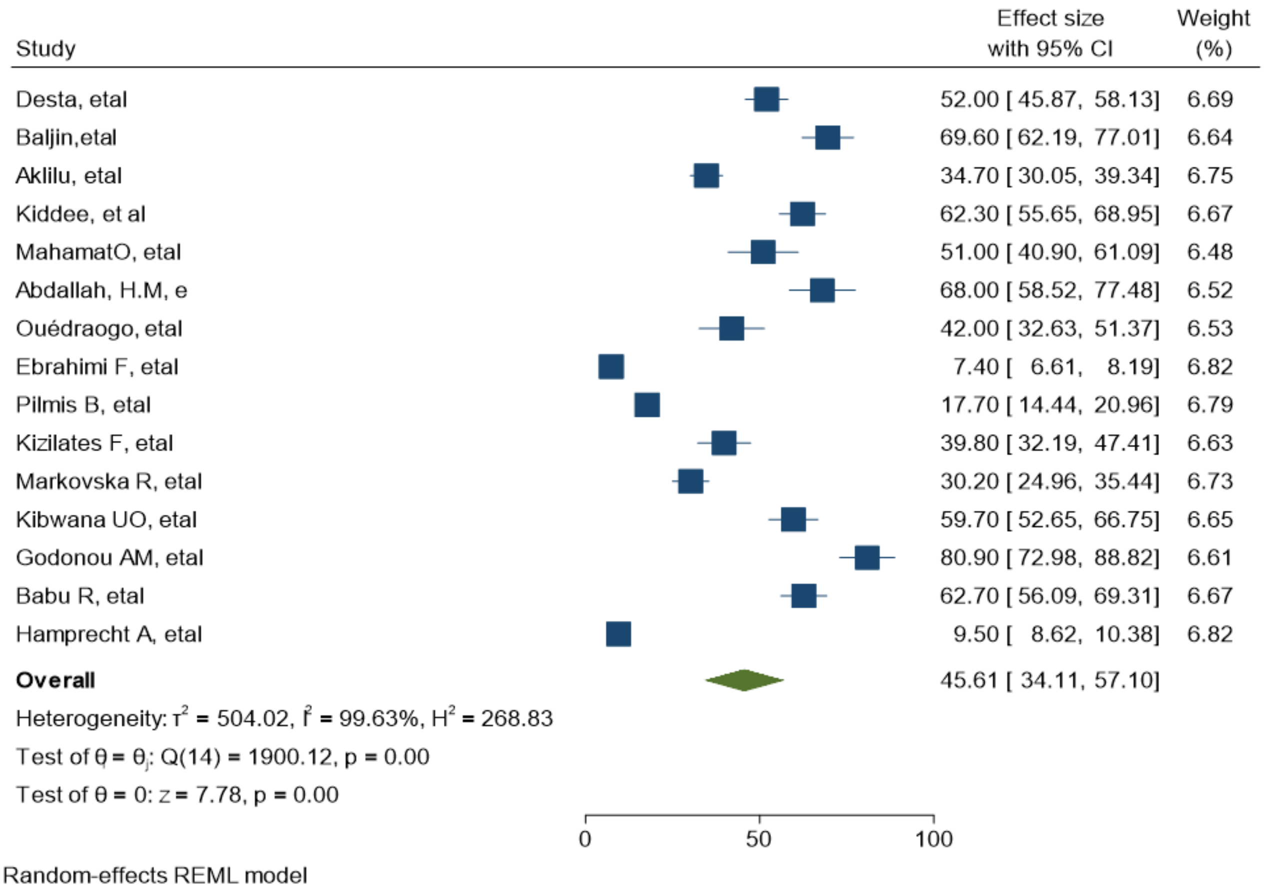

Figure 2. Forest plot for ESBL-PE colonization of hospitalized patients.

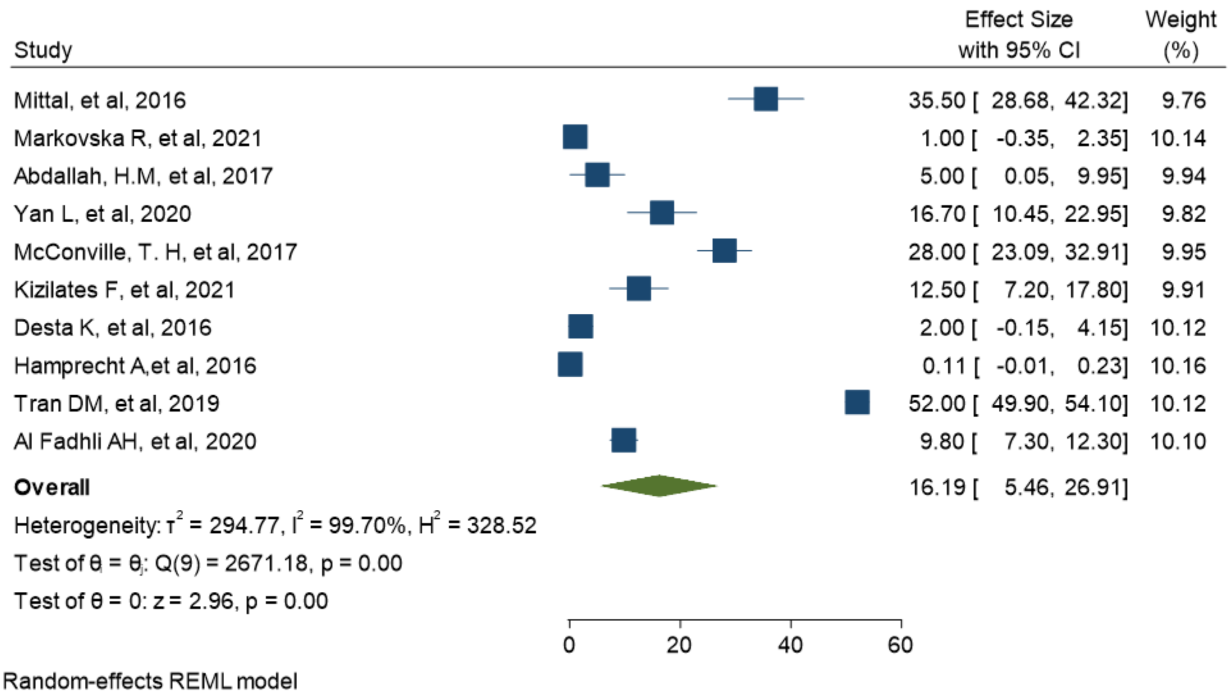

Figure 3. Forest plot for CRE colonization rate of hospitalized patients

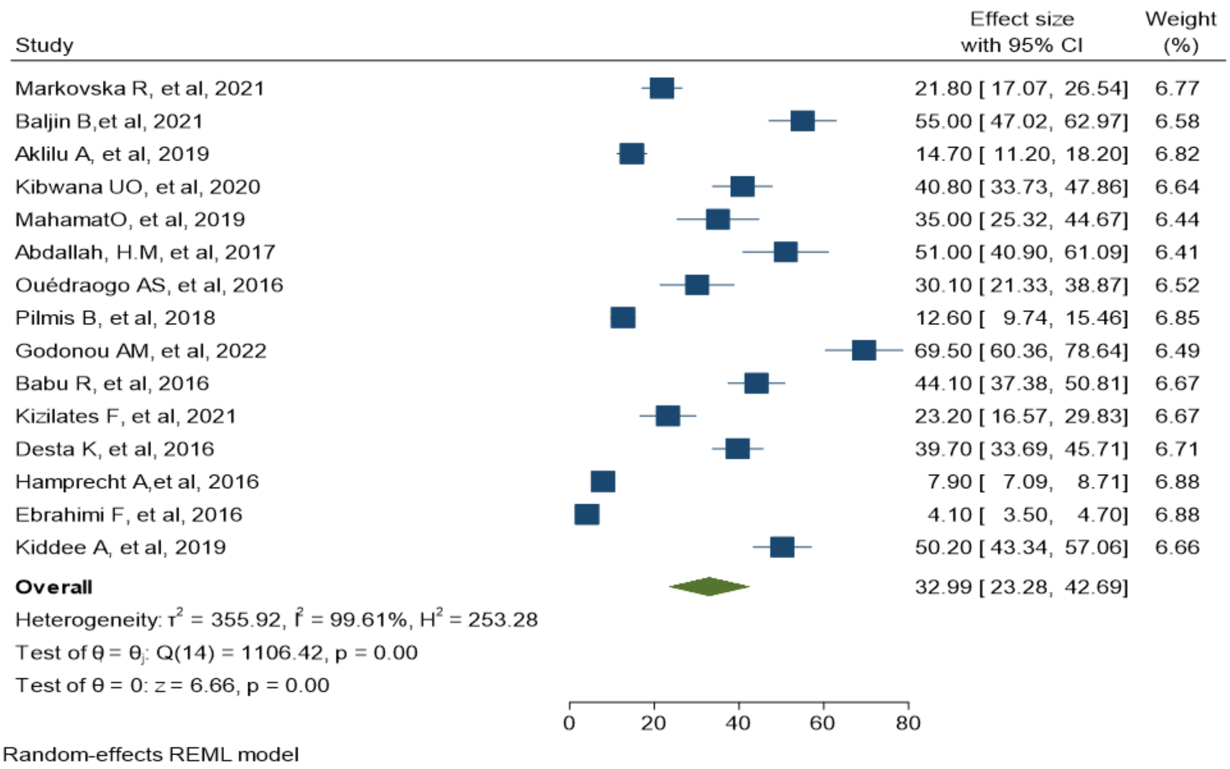

Figure 4. Forest plot for ESBL-producing *E. coli*

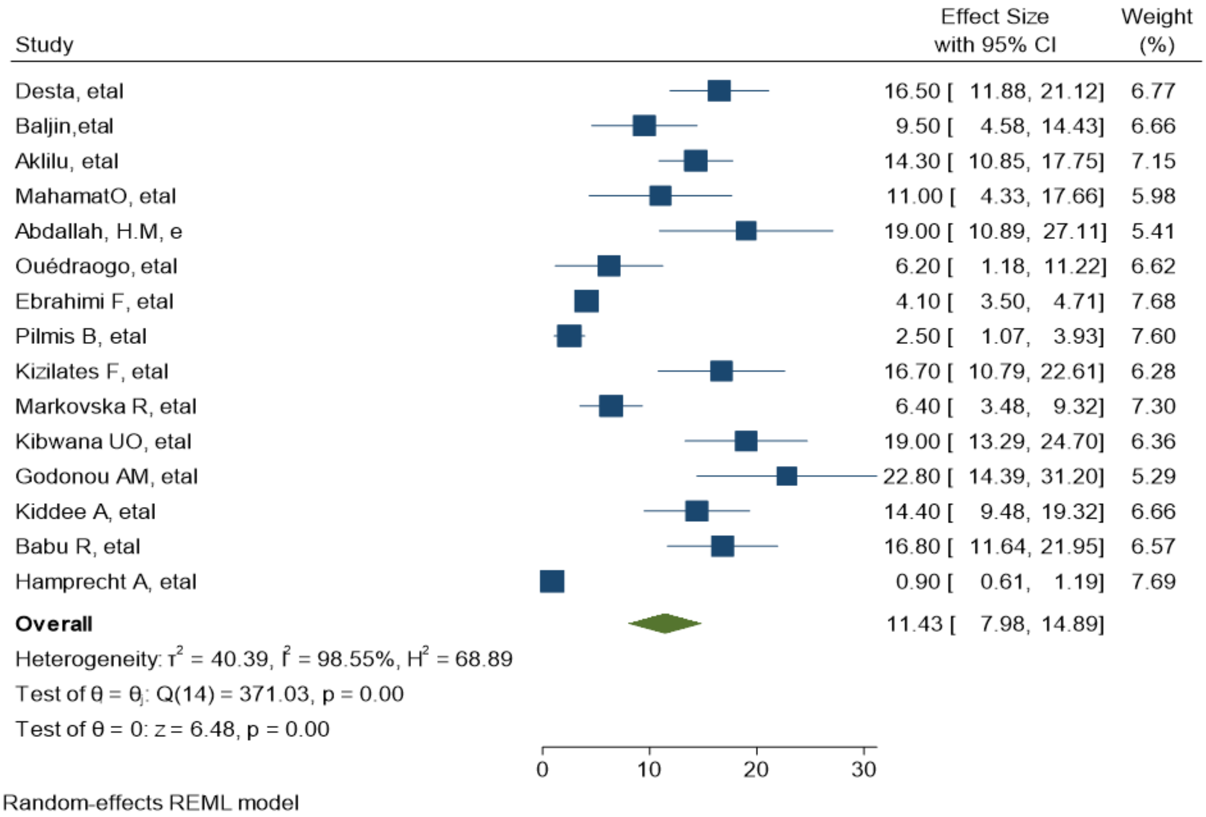

Figure 5. ESBL- producing *K. pneumoniae*

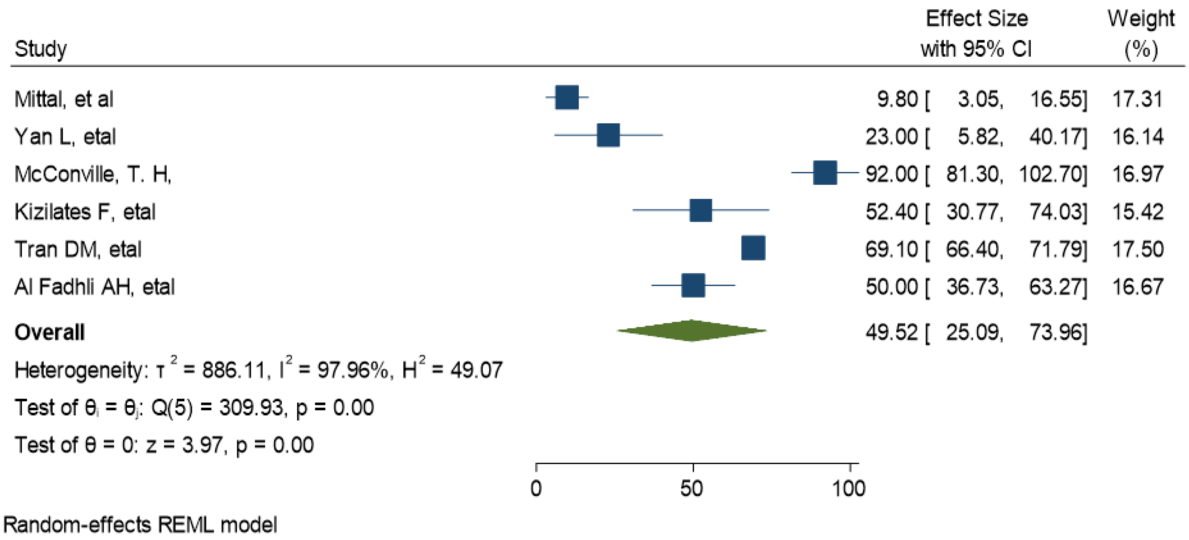

Figure 6. Forest plot showing the estimate of carbapenem producing *K. pneumoniae*

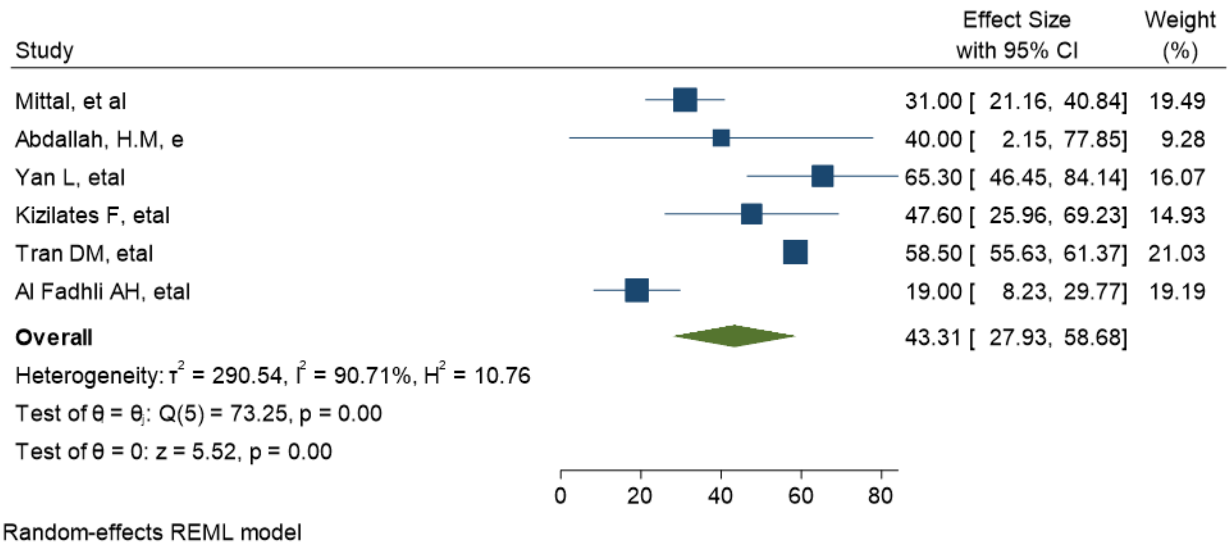

Figure 7. Forest plot showing the estimate of Carbapenem producing *E. coli*

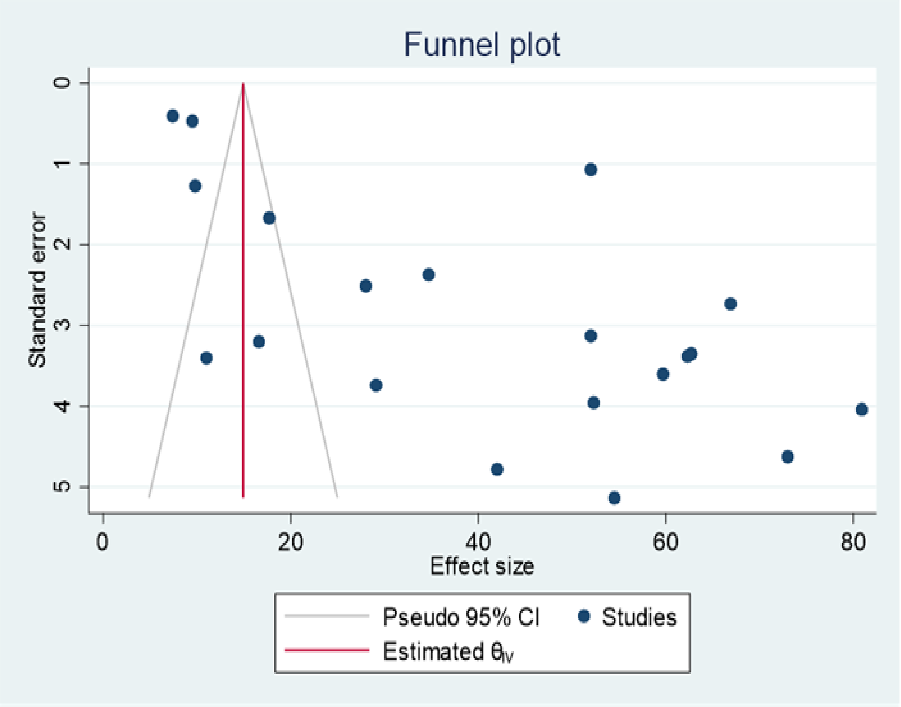

Figure 8. Funnel plot showing publication biases

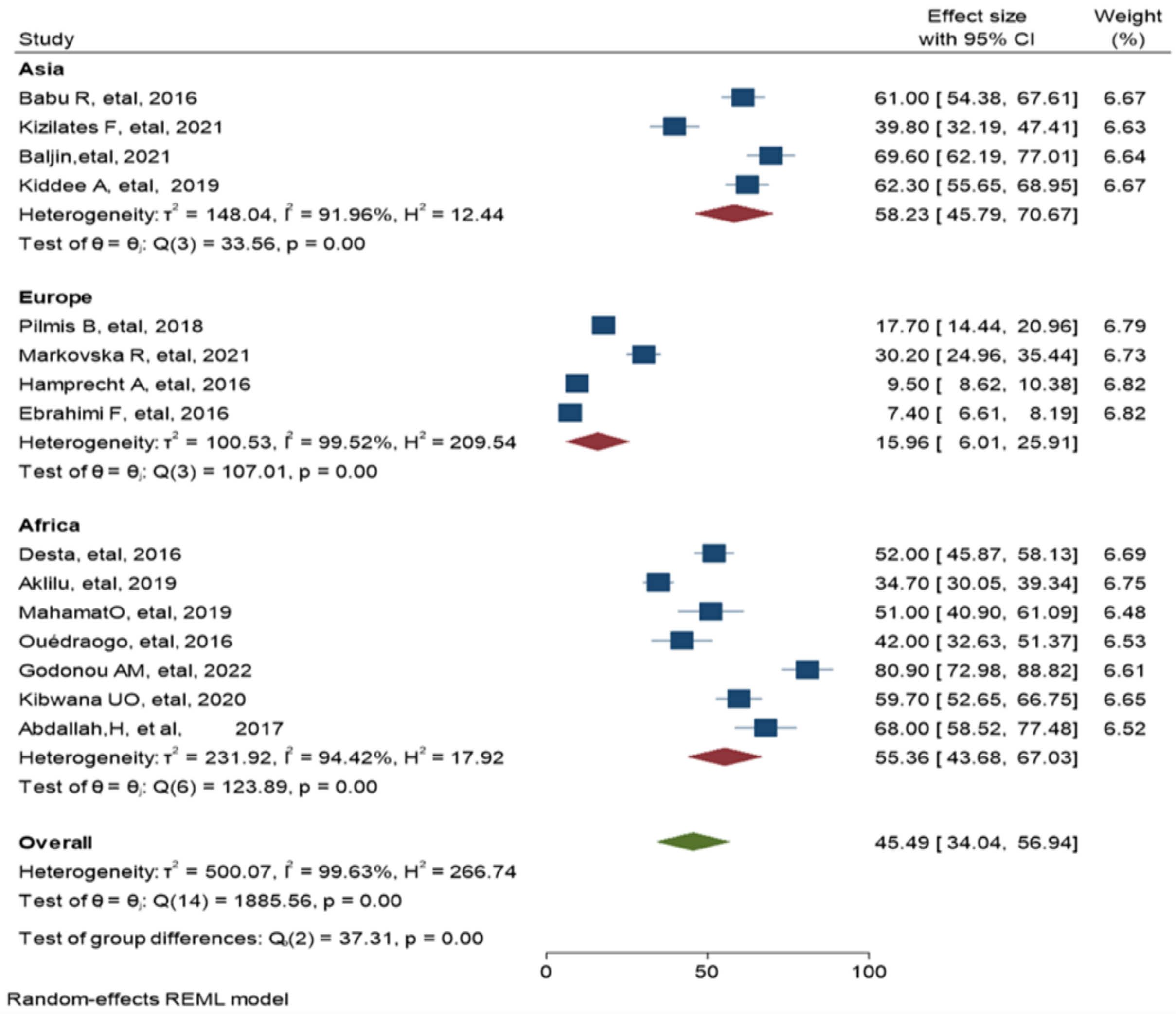

Figure 9. Forest plot showing subgroup analysis of ESBL-PE in Asia, Europe and Africa

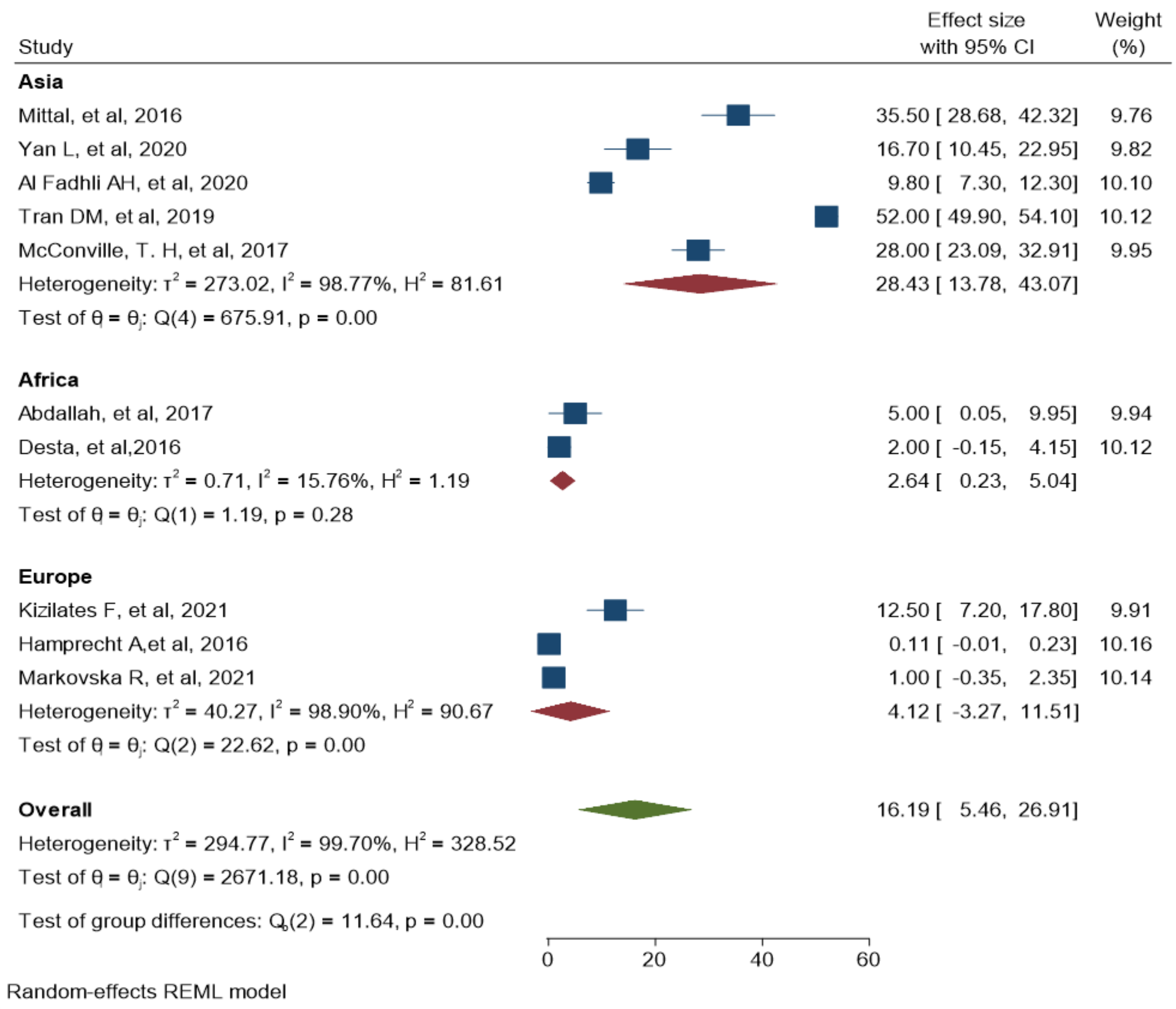

Figure 10. Forest plot of CRE colonization in Asia, Africa and Europe region

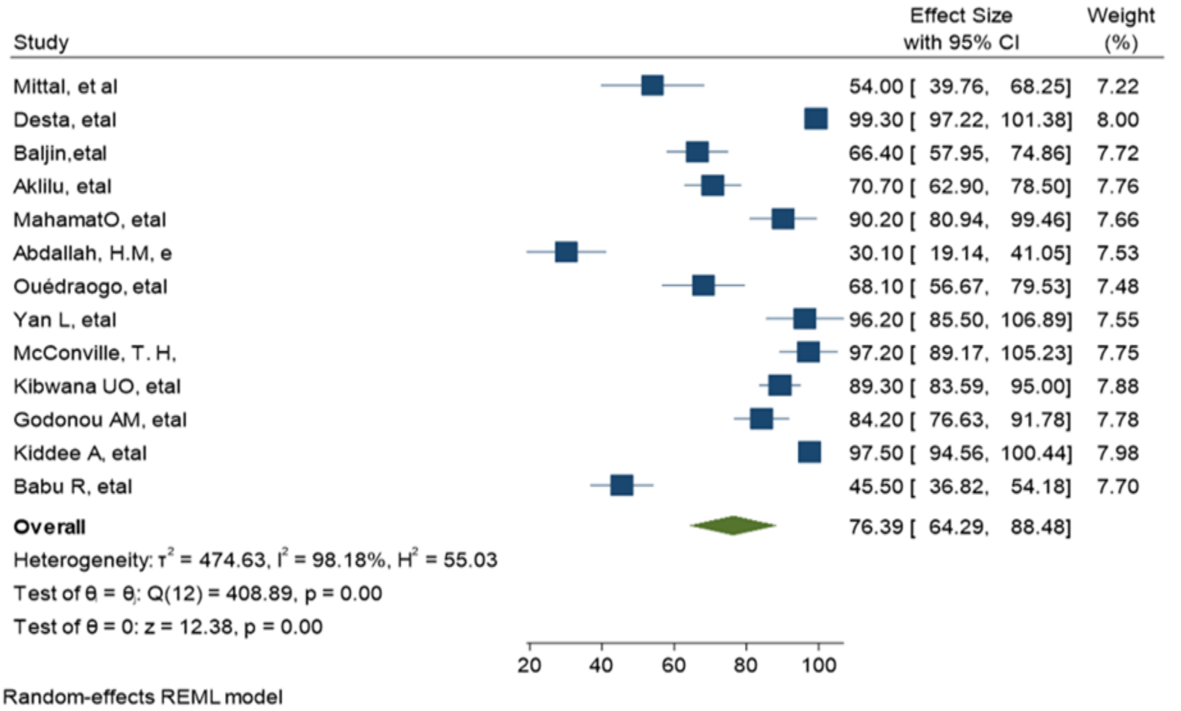

Figure 11. Pooled estimate of Multidrug resistant level.

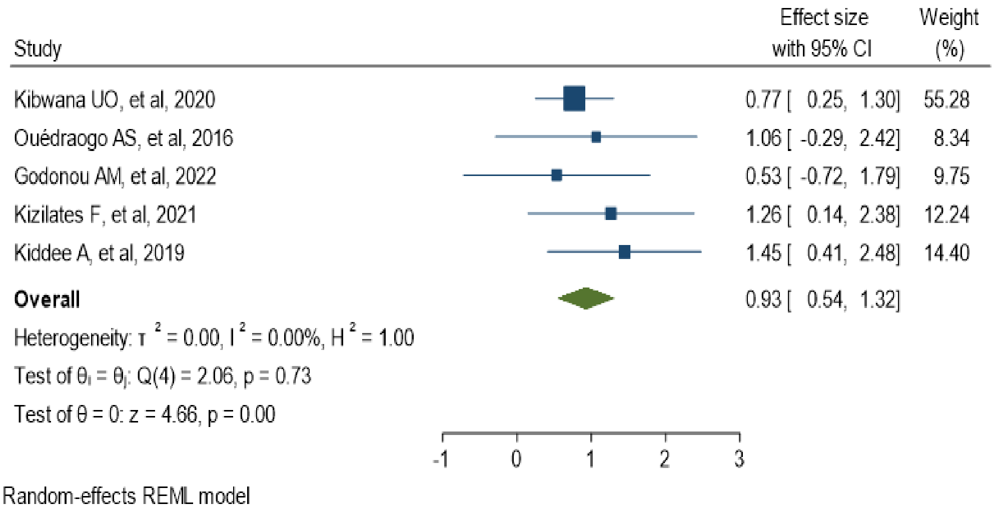

Figure 12. Forest plot of the meta-analysis of ESBL-PE colonization with previous hospitalization

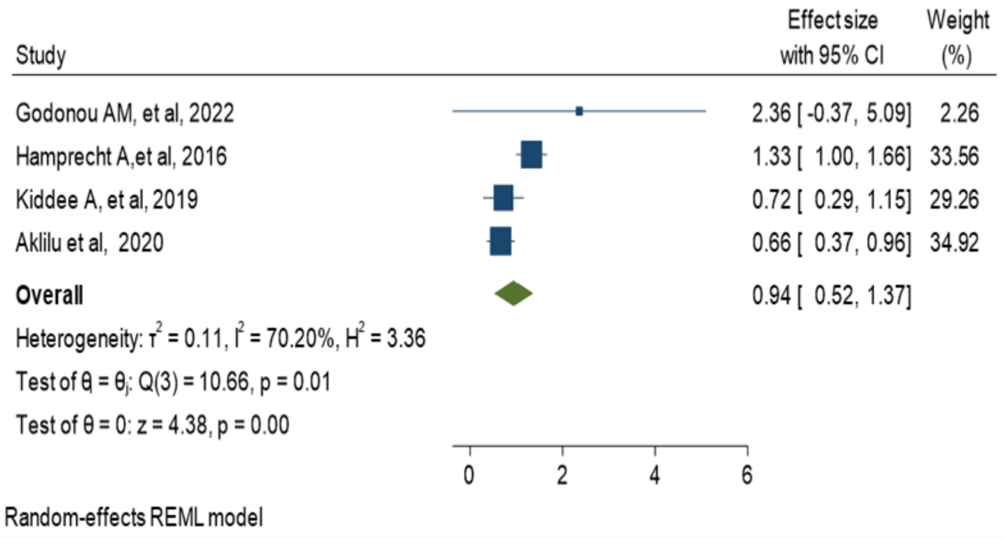

Figure 13. Forest plot for the meta-analysis of hospital stay with ESBL-PE colonization

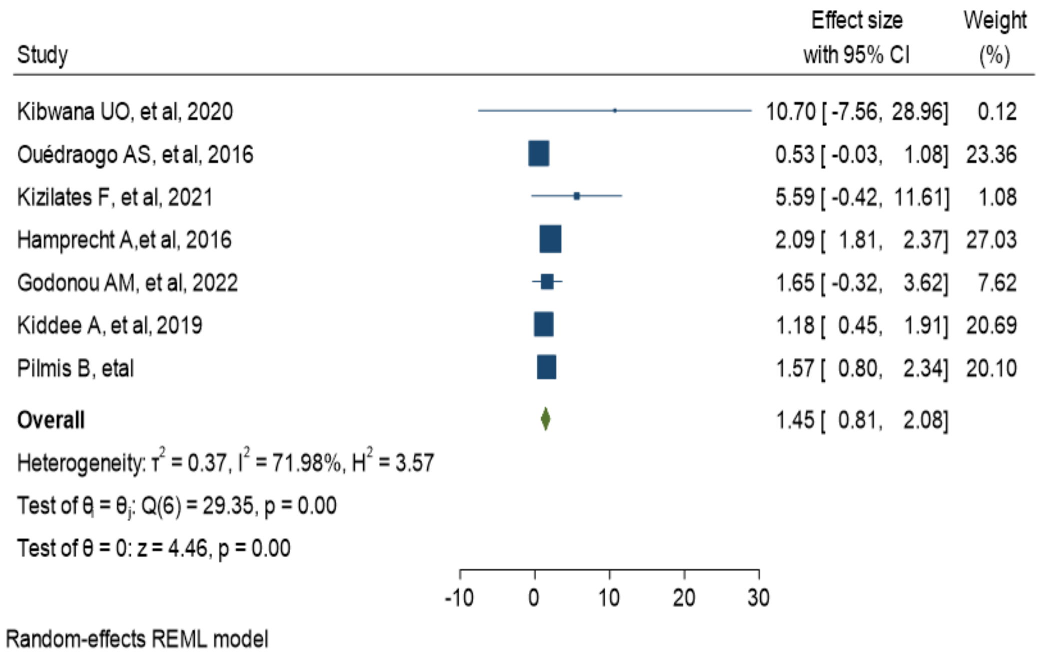

Figure 14. Odds ratio for ESBL colonization and previous antibiotics

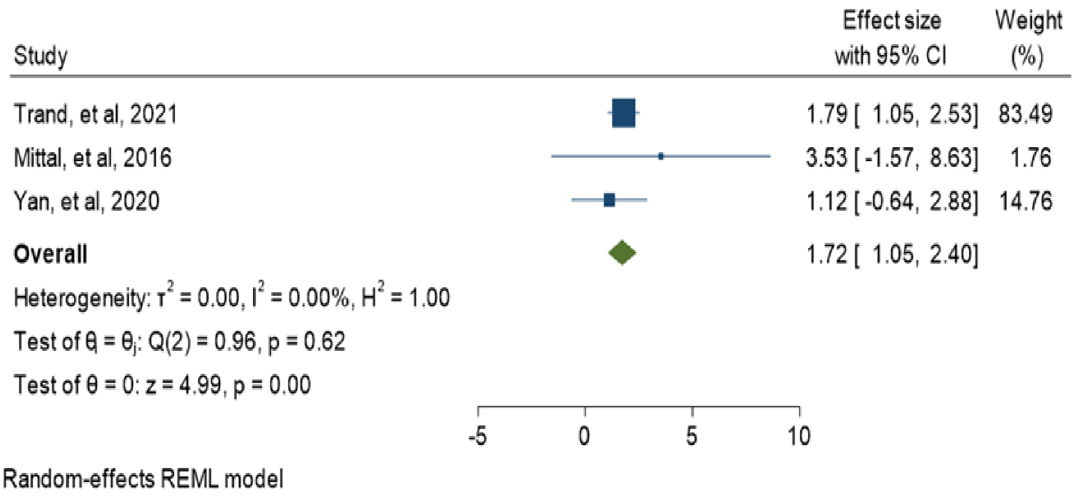

Figure 15. forest plot for carbapenem and CRE colonization

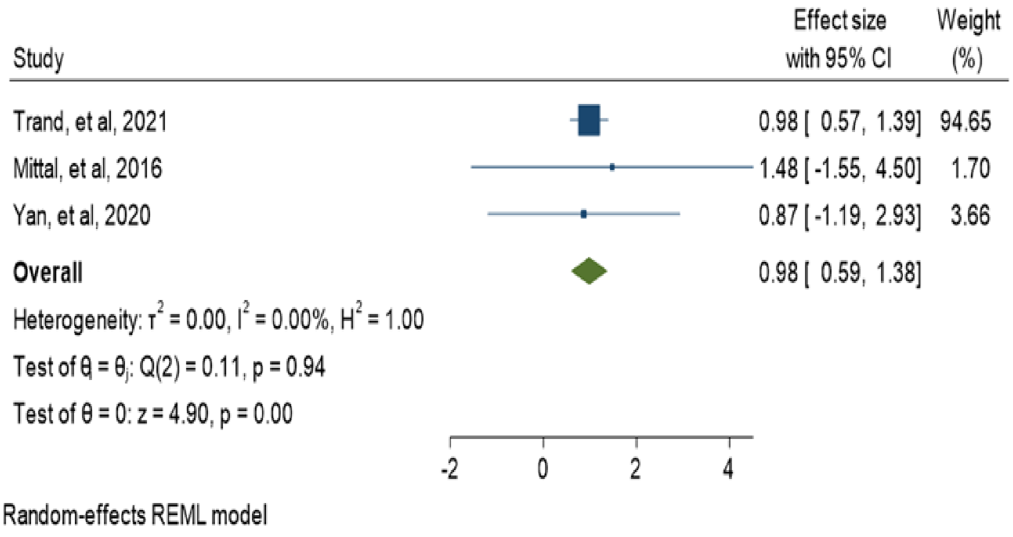

Figure. 16. Forest plot for fluoroquinolone and CRE colonization

Click here to access/download
Supporting Information
S12020 check list.docx
