# Supplementary material for: Colonization with extended spectrum beta-lactamase and carbapenemases producing Enterobacteriaceae among hospitalized patients at the global level: A systematic review and meta-analysis
Source: PLoS One. 2023 Nov 27;18(11):e0293528. doi: 10.1371/journal.pone.0293528 (PMC10681255; doi:10.1371/journal.pone.0293528)
Supplement: Attachment — Submitted filename: comments for consideration.pdf [file pone.0293528.s006.pdf]

Title

Line 1: Colonization with extenede d spectrum beta-lactamase and carbapenemases producing Enterobacteriacae among hospitalized patients at the global level: a systematic review and meta-analysis – double check the spelling of the word extended

Abstract

Line 27 and line 84: Web Science >> Web of Science, and throughout the manuscript

Line 29: carbapenem producing Enterobacteriaceae >> carbapenemase producing Enterobacteriaceae….correct this throughout the manuscript including lines 34, 38, 92

Line 37: E.coli,32.99% (95% CI: 23.28-42.69) and K.pneumoniae, >>> E. coli, 32.99% (95% CI: 23.28-42.69) and K. pneumoniae….be mindful of how the species names are written

Line 61: There are many factors that can facilitate the colonization rate of MDRB in hospitalized patients. Make this list exhaustive and include other factors such as shared hospital environment – sinks, wheelchairs, door knobs (https://doi.org/10.4269/ajtmh.20-1522) and resources (https://doi.org/10.2147/IDR.S303860).

Line 72: lack of resource >> lack of resources

Why use Drug resistance and not antibiotic resistance in the MeSH? Would not the latter return different search results?

Line 96: All studies starting from January1, 2016 to August10, 2022. Would not this be under the inclusion criteria explain why this study period

Line 128: PRISMA 2020 check list was used to indicate where eah >>> rewrite this sentence.

Line 224: pooeld estimated …Check the spelling of the word pooled.

Line 225: Whereas casecontrol >> case-control do the same for line 229

Line 310: This variation might be due to the distribution of bacteria changes over time across countries. This is a vague statement... consider removing it.

Line 314: related with high consumption of beta lactam and carbapenem antibiotic and most CTX-M genes were emerged from this continent and spread to other regions.. Rephrase this sentence please for clarity.

Line 320: considerable report might be due to lack of stewardship and limited resources to prevent….It should be poor antibiotic stewardship and not lack of stewardship
