# Supplementary material for: Colonization with extended spectrum beta-lactamase and carbapenemases producing Enterobacteriaceae among hospitalized patients at the global level: A systematic review and meta-analysis
Source: PLoS One. 2023 Nov 27;18(11):e0293528. doi: 10.1371/journal.pone.0293528 (PMC10681255; doi:10.1371/journal.pone.0293528)
Supplement: Attachment — Submitted filename: review.docx [file pone.0293528.s004.docx]

It is recommended to enter three valid databases that are under review. At least Embase or Scopus should be added.

Which method did you use to categorize the I2 values? Please state the reference

Why didn't you use the Egger and Begg tests for assessing publication bias?

(209): Please report your findings using subgroup analysis on the type of study (cohort, case control or cross sectional)
