# Supplementary material for: Colonization with extended spectrum beta-lactamase and carbapenemases producing Enterobacteriaceae among hospitalized patients at the global level: A systematic review and meta-analysis
Source: PLoS One. 2023 Nov 27;18(11):e0293528. doi: 10.1371/journal.pone.0293528 (PMC10681255; doi:10.1371/journal.pone.0293528)
Supplement: Attachment — Submitted filename: Response to reviewers.docx [file pone.0293528.s005.docx]

**Response to reviewers**

**Journal name:** **PLOS ONE**

**PONE-D-23-02816**: Colonization with extended spectrum beta-lactamase and carbapenemases producing *Enterobacteriaceae* among hospitalized patients at the global level: a systematic review and meta-analysis.

**We would like to say thank you all reviewers and editors, they have seen the paper deeply and forwarded us so valuable comments and suggestions which improves the paper quality in all directions. We have tried to address all the comments and suggestions given by reviewers and editors. Therefore, the manuscript is revised accordingly your requirements and all revisions can be checked in the manuscript easily since all the necessary changes are shaded in blue. In addition, all authors have revised the manuscript after addressing reviewer’s comments and suggestions and we have made significant improvement on the quality, readability and clarity of the manuscript.**

**Review Comments to the Author**

**Reviewer #1**: -The Line 2. To replace the carbapenem with carbapenemases producing Enterobacteriaceae in the title to be more organized

We thank you, we have modified it as carbapenemases at the title section (page 1)

-In line 25, mention carbapenemases instead of carbapenem to describe the study enzymes uniformly.

Thank you so much we have replaced it with carbapenemases at abstract section (page 2, line 26).

-In line 47, CRE is deleted and replaced by MDRB

We thank you we have corrected it as MDRB (page 3, line 47)

-In line 75, The following references must be used to support the idea:-

Al-Qaysi, A. K., Al-Ouqaili, . M. T. & Al-Meani, S. A. (2020). Ciprofloxacin- and gentamicin-mediated inhibition of Pseudomonas aeruginosa biofilms is enhanced when combined with the volatile oil from Eucalyptus camaldulensis. SRP, 11 (7), 98-105.

We thank you very much for suggesting such important paper. We have included the article at the introduction part (page 5, line 71-72).

In line 135, I think not to write in the world but most of the world where there are studies from the countries which are not cited. Thus, we will provide you with some important and so related works to create a study more comprehensive

Thank you, we modified it as indicated in ( Page 8, line 131-132):

All age groups, gender (male/female) and any ethnic groups live in different countries in most of the world were included in this study.

-To table 2, Please use the following references and refer to the works in the following table and the country. This is important for your study to be more comprehensive and informative:-

1-AL-KUBAISY SH, HUSSEIN, RA, AL-OUQAILI, MTS. (2020). Molecular Screening of Ambler class C and extended spectrum β-lactamases in multi-drug resistant Pseudomonas aeruginosa and selected species of Enterobacteriaceae. International Journal of Pharmaceutical Research | Jul - Sep 2020 | Vol 12 | Issue 3.

2-Al-Ouqaili, MTS, Khalaf EA, Al-Kubaisy SH. (2020). DNA Sequence Analysis of BlaVEB Gene Encoding Multi-drug Resistant and Extended-spectrum β-lactamases Producer Isolates of Enterobacteriaceae and Pseudomonas aeruginosa. The Open Microbiology Journal, 4: 40-47.

3-Al-Ouqaili, MTS, Al-Taei, SA, Al-Najjar A. Molecular Detection of Medically Important Carbapenemases Genes Expressed by Metallo-β-lactamase Producer Isolates of Pseudomonas aeruginosa and Klebsiella pneumoniae. Asian Journal of Pharmaceutics • Jul -Sep 2018 (Suppl ) • 12 (3) | S991.

4-Khalaf, EA, Al-Ouqaili, MTS. Molecular detection and sequencing of SHV gene encoding for extended-spectrum β-lactamases produced by multidrug resistance some of the Gram-negative bacteria. International Journal of Green Pharmacy • Oct-Dec 2018 (Suppl) • 12 (4) | S910-S918.

We thank you so much. These are interesting studies from different clinical specimens, however our main objective is to review ESBL-PE and CRE from stool specimens. Therefore, we have agreed to include these studies in the discussion part (page 19, line280-281.)

-In line 272, It is important to refer to the following reference: Al-Ouqaili, MTS., Jal'oot, AS., Badawy, AS. Identification of an OprD and bla(IMP) Gene-mediated Carbapenem Resistance in Acinetobacter baumannii and Pseudomonas aeruginosa among Patients with Wound Infections in Iraq. Asian Journal of Pharmaceutics., volume 12, Issue 3, 2019 Page S959-S965-

We thank you, and we have included this study in the discussion part (page 19, line 281).

-In the conclusion statement, particularly, line 388, Please do add to line 388 as follow: - antibiotic treatment and the duration in addition to a long stay in the hospital

Thank you so much, and we have added based on your suggestion as follows:

Risk factors such as previous antibiotic treatment and the duration in addition to a long stay in the hospital stay were associated with ESBL-PE and CRE colonization (page 25, line 401-403).

Reviewer #2: It is recommended to enter three valid databases that are under review. At least Embase or Scopus should be added.

Thank you for pick this comment up, however we agreed to write this issue as a limitation as indicated in (page 25, line 397).

Which method did you use to categorize the I2 values? Please state the reference

Thank you for this comment. The heterogeneity categorization was based on the Higgins JP, Thompson SG, Deeks JJ, Altman DG. Measuring inconsistency in meta-analyses. Bmj. 2003;327(7414):557-60 (page 11, line 158-159).

Why didn't you use the Egger and Begg tests for assessing publication bias?

Thank you very much for this comment and we have included the egger test for publication bias as indicated at the result section (page 11, line 160) and Table 4.

(209): Please report your findings using subgroup analysis on the type of study (cohort, case control or cross sectional).

Thank you so much, and we have analyzed the subgroup analysis for the study design as indicated at the result section (page 15, line 222-231) and discussion section (line 350-355).
