# Supplementary material for: Colonization with extended spectrum beta-lactamase and carbapenemases producing Enterobacteriaceae among hospitalized patients at the global level: A systematic review and meta-analysis
Source: PLoS One. 2023 Nov 27;18(11):e0293528. doi: 10.1371/journal.pone.0293528 (PMC10681255; doi:10.1371/journal.pone.0293528)
Supplement: Attachment — Submitted filename: Response to reviewers.docx [file pone.0293528.s007.docx]

**Response to reviewers**

**Journal name:** **PLOS ONE**

**PONE-D-23-02816**: Colonization with extended spectrum beta-lactamase and carbapenemases producing *Enterobacteriaceae* among hospitalized patients at the global level: a systematic review and meta-analysis.

**First of all, we would like to say thank you all reviewers and editors, they have seen the paper deeply and forwarded us so valuable comments and suggestions which improves the paper quality in all directions.**

**All comments and suggestions given by reviewers and editors are taken well and incorporated point by point. For your verification, we just put editor and reviewers comment as they gave us and all our response what we have for each point are indicated below each comment (shaded in blue). Hence, the manuscript is revised accordingly your requirements and all revisions can be checked in the manuscript easily since all the necessary changes are shaded in blue. In addition, all authors have revised the manuscript after addressing reviewer’s comments and suggestions and we have made significant improvement on the quality, readability and clarity of the manuscript.**

**Review Comments to the Author**

Reviewer #3

Title

Line 1: Colonization with exteneded spectrum beta-lactamase and carbapenemases producing

Enterobacteriacae among hospitalized patients at the global level: a systematic review and metaanalysis

– double check the spelling of the word extended

We thank you so much. We have corrected it as extended (line 1), and we have also corrected in other sections as well.

Abstract

Line 27 and line 84: Web Science >> Web of Science, and throughout the manuscript

We thank you and we have corrected it as Web of Science as indicated in (line 27 and 84).

Line 29: carbapenem producing Enterobacteriaceae >> carbapenemase producing

Enterobacteriaceae….correct this throughout the manuscript including lines 34, 38, 92

We thank you very much, and we have corrected throughout the document including (line 34, 38, 92) as carbapenemase producing *Enterobacteriaceae*.

Line 37: E.coli,32.99% (95% CI: 23.28-42.69) and K.pneumoniae, >>> E. coli, 32.99% (95%

CI: 23.28-42.69) and K. pneumoniae….be mindful of how the species names are written

We thank you and we have corrected it in (line 37) as *E. coli*, 32.99% (95% CI:23.28-42.69) and *K. pneumoniae*, 11.43% (95% CI:7.98-14.89) throughout the document.

Line 61: There are many factors that can facilitate the colonization rate of MDRB in

hospitalized patients. Make this list exhaustive and include other factors such as shared

hospital environment – sinks, wheelchairs, door knobs (https://doi.org/10.4269/ajtmh.20-

1522) and resources (<https://doi.org/10.2147/IDR.S303860>).

We thank you very much and we have modified based on your suggestion.

There are many factors that can facilitate the colonization rate of MDRB in hospitalized patients: such as low hygiene practice, improper antibiotic usage, prolonged hospitalization, implanted materials, and sharing a single room with high carrier patients including door knobs, wheelchairs and sinks have been associated (page 4, line 61-64). (<https://doi.org/10.2147/IDR.S303860>), https://doi.org/10.1016/j.jhin.2023.06.020 and <https://doi.org/10.1017/ice.2018.359>.

Line 72: lack of resource >> lack of resources

We thank you and we corrected it as lack of resources (page 5 line 73).

Why use Drug resistance and not antibiotic resistance in the MeSH? Would not the latter

return different search results?

We thank you. Both terms gave us similar results, but we preferred to use Drug resistance because it gave us more results.

Line 96: All studies starting from January1, 2016 to August10, 2022. Would not this be under

the inclusion criteria explain why this study period

Thank you for the valuable comment. In the revised manuscript, we have included the study period in the inclusion criteria. We selected this study period to have recently updated global data on the raised issue (page 6, line 103).

Line 128: PRISMA 2020 check list was used to indicate where eah >>> rewrite this sentence.

We thank you, and we have corrected it. as PRISMA 2020 check list was used to indicate detail descriptions found as shown in the supplementary file (see S1 2020 check list) (line 129-130).

Line 224: pooeld estimated …Check the spelling of the word pooled.

We thank you we have corrected it as pooled indicated in line 225.

Line 225: Whereas casecontrol >> case-control do the same for line 229

We thank you so much. We have corrected it as case-control in line 226 and 230.

Line 310: This variation might be due to the distribution of bacteria changes over time across

countries. This is a vague statement... consider removing it.

We thank you so much. We have removed it.

Line 314: related with high consumption of beta lactam and carbapenem antibiotic and most CTX-M genes were emerged from this continent and spread to other regions. Rephrase this

sentence please for clarity

Thank you so much. We have modified the sentence as indicated in (page 20, line 313-315)

This high prevalence might be related to the high consumption of beta-lactam antibiotics, leading to the emergence of plasmid-mediated resistance to beta-lactam antibiotics.

Line 320: considerable report might be due to lack of stewardship and limited resources to

prevent….It should be poor antibiotic stewardship and not lack of stewardship

We thank you and we have corrected it based on your suggestion (line319-321). The reason for this considerable report might be due to poor antibiotic stewardship and limited resources to prevent and control the spread of multidrug-resistance.
